# Multi-Scale Entropy-Gated Deep Quantile Learning via EntropyFusion-VaR: Tail-Risk Forecasting for U.S. Energy-Sector Equities

**DOI:** 10.3390/e28101165

**Published:** 2026-10-21

**Authors:** Adila Osman, Yangxin Wang, Yuanyuan Wang

**Affiliations:** 1 School of Finance, Xinjiang University of Finance and Economics, Urumqi 830012, China; 2 School of Economics and Business Administration, Central China Normal University, Wuhan 430079, China; 3 Bangor College, Central South University of Forestry and Technology, Changsha 410004, China; h20190192@csuft.edu.cn

**Keywords:** multi-scale entropy, transfer entropy, deep quantile learning, EntropyFusion-VaR, tail risk, U.S. energy equities, entropic risk-sensitive optimization

## Abstract

Forecasting extreme-tail risk in energy equities involves temporal-memory mismatch, quantile crossing, and scarce extreme observations. This study proposes a multi-scale entropy-gated deep quantile learning model, EntropyFusion-VaR. Weighted permutation entropy (WPE) characterizes local ordinal disorder, Rényi entropy describes concentration in the full binned return distribution, and transfer entropy (TE) describes directional predictive information. These state features drive a gate that coordinates a causal dilated TCN and a Patch Transformer. An incremental cumulative output layer ensures ordered joint quantiles at the 1%, 2.5%, and 5% levels. An exponential aggregation of pinball losses gives greater optimization weight to large forecast errors, while a differentiable coverage penalty regularizes exception frequencies. Experiments on EOG Resources (EOG), Marathon Petroleum (MPC), and Schlumberger (SLB) show lower reported average tail loss and zero quantile crossing. The model attains the best mean rank at each studied horizon and ranks first in eight of nine stock–horizon combinations. The evidence supports state-dependent tail-risk forecasting for these three U.S. energy-sector equities, with coverage assessed separately from loss improvement.

## 1. Introduction

Amid the combined shocks of pronounced global macroeconomic turbulence, recurrent geopolitical conflicts, and the worldwide low-carbon energy transition, energy equity markets have exhibited exceptionally high price volatility and complex extreme-risk characteristics. As a core metric in financial risk measurement, Value at Risk (VaR) quantifies the maximum potential loss that a portfolio may incur over a specified time horizon at a given confidence level. Accurate VaR forecasting for energy equities, particularly for extreme-tail risk at the 1% confidence level, is strategically important for investors seeking to optimize asset allocation and prevent severe drawdowns, as well as for regulators formulating sound capital-adequacy requirements. However, energy-market price fluctuations are often accompanied by abrupt information shocks and pronounced asymmetric downside risk. Rapidly changing market microdynamics therefore pose unprecedented challenges to conventional risk-measurement paradigms.

Over decades of financial risk-management practice and academic research, numerous classical models have been developed. Yet their limitations become increasingly difficult to overcome when they are applied to extreme outcomes in energy equity markets and to complex small-sample settings. Traditional nonparametric methods such as Historical Simulation (HS) respond to tail extremes with a severe lag in highly volatile markets (the so-called “ghost effect”) and lack smoothness. Parametric volatility models represented by the GARCH family, meanwhile, often rely heavily on particular heavy-tailed distributional assumptions or linear specifications of volatility dynamics, making them unable to fully characterize the highly nonlinear and nonstationary dynamics displayed by financial assets under extreme shocks. In recent years, deep-learning benchmarks such as Long Short-Term Memory (LSTM) networks and Temporal Convolutional Networks (TCNs) have performed well in forecasting conditional means or volatility. Nevertheless, they remain constrained by three major bottlenecks when the focus shifts to “extreme VaR forecasting”: first, a single architecture struggles to accommodate both short-run local jumps and long-run market memory; second, jointly outputting multiple tail quantiles readily gives rise to “Quantile Crossing”, which violates financial logic; and third, crash observations are extremely scarce in the far tail, while conventional loss functions lack distributional robustness, leaving the model vulnerable to overfitting or distorted extreme-value forecasts.

To address these challenges, this study proposes a multi-scale entropy-gated deep quantile learning model (EntropyFusion-VaR), which integrates information-theoretic multi-scale state measures with deep temporal networks to form a comprehensive framework for forecasting the tail risk of energy equities. Its core architecture has four dimensions. First, a dynamic entropy gate and a dual-expert system incorporate multi-scale weighted permutation entropy (WPE), Rényi entropy, and transfer entropy (TE) into the network. According to the prevailing market state, the gate dynamically adjusts the predictive weights assigned to a local expert (a causal dilated TCN) and a long-memory expert (Patch Transformer), enabling adaptive coordination between short-run shocks and long-term memory. Second, a Risk-State Skip injects key conventional financial prior signals, such as volatility, directly into deeper network layers through a residual connection, thereby preventing information loss. Third, a non-crossing multi-quantile output mechanism uses an incremental cumulative mapping to guarantee that the predicted quantiles satisfy *Q*0.01 ≤ *Q*0.025 ≤ *Q*0.05. Fourth, an entropic robust quantile-loss function assigns greater weight to large errors and incorporates coverage calibration, enhancing sensitivity to extremes while restraining excessively conservative forecasts.

The empirical analysis focuses on EOG, MPC, and SLB, which span upstream exploration and production, downstream refining, and oilfield services. The reported comparisons assess average forecast loss, coverage, and joint-quantile consistency within this sample. For risk managers holding these equities, ordered dynamic quantiles provide coherent risk boundaries, while gate weights offer an auxiliary view of how the model allocates emphasis between temporal experts. The practical contribution is an integrated forecasting and diagnostic framework whose component effects can be assessed against the corresponding loss, crossing, and ablation evidence.

## 2. Literature Review

Energy equities are jointly affected by commodity prices, macroeconomic policy, geopolitics, and corporate operating leverage, and their return distributions typically exhibit volatility clustering, heavy tails, and regime shifts. Accordingly, the literature has gradually expanded from conditional-volatility modeling to deep quantile forecasting and information-theoretic state identification. This review is organized around three strands: tail-risk measurement and backtesting, deep learning and joint-quantile forecasting, and entropy-driven identification of market states. These strands jointly define the research contribution of EntropyFusion-VaR.

The empirical motivation for studying non-Gaussian returns has a long history. Mandelbrot [1] challenged Gaussian descriptions of speculative price changes, and Mantegna and Stanley [2] examined scaling in an economic index, including differences between the central distribution and its tails. Gopikrishnan et al. [3] documented return-distribution scaling over several time scales. These studies motivate attention to large financial fluctuations without imposing a universal distributional law on the assets considered here.

Wątorek et al. [4] examine return distributions across asset classes, time scales, and the COVID-19 regime, providing more recent evidence on variation in tail behavior. Heavy tails describe departures from Gaussian tail decay; a specific power law requires separate tail estimation. Sample kurtosis is a finite-sample summary, rather than an estimator of an asymptotic tail exponent. This literature concerns distributional stylized facts, whereas the present forecasting task estimates risk boundaries conditional on historical information at daily and multi-day horizons. It motivates the task; the proposed architecture is evaluated by its own out-of-sample comparisons.

### 2.1. VaR, Tail-Risk Modeling, and Out-of-Sample Backtesting

Traditional VaR research has developed primarily along two paths: conditional-volatility modeling and dynamic-quantile modeling. GARCH and GJR-GARCH, which capture volatility clustering and shock asymmetry, respectively, are classical benchmarks for financial risk forecasting [5,6]. CAViaR instead models conditional quantiles recursively, allowing the risk boundary to adjust with lagged VaR and return shocks [7]. The former relies on specifications of the volatility equation and innovation distribution, whereas the latter is constrained by a relatively parsimonious parametric form. Both may consequently be misspecified under structural breaks, high-dimensional information, and multi-scale dependence.

The validity of VaR must also be assessed using coverage tests. The Kupiec test examines whether the exception frequency is consistent with the nominal probability, while the Christoffersen test further identifies the clustering of exceptions [8,9]. Passing coverage tests, however, does not imply that a model adequately captures the magnitude of tail losses. Liu and Wang show axiomatically that VaR and ES reflect different aspects of tail information [10]. Risk evaluation should therefore jointly report coverage, exception dynamics, quantile loss, and loss severity.

Recent research has increasingly emphasized the joint identification and robust backtesting of VaR and ES. Merlo et al. embed joint-quantile regression in asset allocation, while Bayer and Dimitriadis propose regression-based ES backtests [11,12]. Geenens and Dunn use a nonparametric Copula to reduce distributional misspecification, and Kuang documents pronounced regime differences in crude-oil tail risk during the financial crisis and the pandemic [13,14]. These findings indicate that the risk boundaries of energy assets vary jointly with market regimes, asset-dependence structures, and external shocks.

Multi-horizon forecasting imposes further requirements on dynamic specifications. Zhang et al. find that horizon aggregation, jumps, and forward-looking moments alter the performance of multi-period VaR and ES, whereas Dierkes et al. show that different tail-risk measures are not equivalent in either statistical identification or economic information [15,16]. Christensen et al. demonstrate that machine learning can extract nonlinear information from realized volatility and an expanded set of covariates [17], but such improvements have risk-management value only when they are translated into well-calibrated tail quantiles. Differences in model loss should also be subjected to out-of-sample significance testing, for example with the Diebold–Mariano test [18]. Overall, the econometric literature establishes the definitions and backtesting discipline of risk measurement but still struggles to address high-dimensional nonlinearity, multi-scale dependence, and rapid regime switching simultaneously.

### 2.2. Deep Learning, Joint-Quantile Forecasting, and Non-Crossing Constraints

Deep learning advances VaR forecasting from prespecified low-dimensional recursive equations to data-driven feature extraction. Petneházi combines causal dilated convolution with quantile loss, while Kakade et al. integrate volatility priors and gated memory through a GARCH-LSTM architecture [19,20]. Convolutional structures are effective at extracting local shocks, whereas recurrent structures are suited to preserving longer dependencies. A single backbone, however, generally has to use the same parameterization as a compromise across tranquil periods, jump episodes, and persistent drawdowns.

Generative and probabilistic networks further attempt to learn the entire risk distribution. Encoded VaR uses a variational autoencoder to generate asset-risk scenarios, while DeepVaR evaluates portfolio risk with a probabilistic deep network [21,22]. These methods reduce the need for explicit distributional assumptions, but their tail quality depends heavily on the extent to which the training sample covers extreme regimes. If low-probability regions are over-smoothed, the model may still systematically underestimate VaR.

Direct deep quantile regression provides a more explicit optimization objective. Chronopoulos et al. show that deep quantile models can accommodate high-dimensional and mixed-frequency information, while Wang et al. further combine GAN-based scenario generation with joint VaR and ES forecasting [23,24]. Qiu et al. retain information on market evolution through a stateful recurrent network, whereas Buczyński and Chlebus embed a gating mechanism in the conditional-variance recursion of GARCHNet [25,26]. These findings support nonlinear tail modeling, but they also suggest that compressing local jumps and long-term memory into the same hidden state may intensify training conflicts.

Jointly outputting multiple risk levels may also generate quantile crossing. Moon et al. learn multiple conditional quantiles through a joint neural network, Qi et al. address conditional-quantile forecasting from a distributionally robust perspective, and Shen et al. estimate non-crossing quantile processes using deep ReQU networks and penalization [27,28,29]. Simply summing multiple pinball loss terms therefore does not automatically ensure monotonicity; order constraints must be explicitly encoded in the output mapping or optimization process. Shin and Jung further prevent curve crossing through constrained deep support-vector quantile regression [30], indicating that structural constraints generally preserve the shape of the conditional distribution more effectively than ex post sorting.

The information set used for risk forecasting has also expanded from price series to model combinations and textual data. Wang et al. use regularized combinations to reduce single-model misspecification, Cao et al. introduce textual information into a hybrid deep network, and Song et al. extract investor sentiment with BERT and Transformer models [31,32,33]. High-dimensional information, however, does not inherently confer tail robustness and may remain vulnerable to timing mismatches, data snooping, and overfitting of extreme observations. Reviews of the field note that financial deep learning now encompasses convolutional, recurrent, attention-based, and generative architectures, but model comparisons are often affected by differences in data splitting and benchmark strength, while uncertainty quantification remains underdeveloped [34,35]. A more defensible direction is therefore not to keep increasing the complexity of a single network, but to dynamically combine experts with distinct temporal-scale preferences according to market conditions.

Loukeris et al. [36] extend neural financial modeling through the Optimal Portfolio Selection Intelligence (OPSI) framework, comparing radial-basis-function and neuro-genetic models with time-lag recurrent networks, multilayer perceptrons, and Bayesian logistic regression. Their focus on corporate evaluation and portfolio selection places recurrent memory within a broader financial decision framework. The time-lag recurrent networks considered there belong to the recurrent family and are distinct from the GARCH-LSTM architecture in [20]. EntropyFusion-VaR targets jointly ordered conditional tail quantiles and evaluates them through forecasting loss and coverage backtests.

### 2.3. Entropy-Driven Market-State Identification and the Positioning of This Study

Entropy provides a characterization of market conditions that differs from variance. The permutation entropy proposed by Bandt and Pompe measures the dynamic complexity of a univariate series through ordinal patterns [37], whereas the transfer entropy proposed by Schreiber identifies the directional incremental information that a source series provides about the future state of a target series [38]. The two concepts correspond, respectively, to internal order and cross-market information transmission, making them suitable for characterizing situations in which energy equities are simultaneously exposed to changes in their own volatility and to the diffusion of external risk.

Multi-scale evidence indicates that crises alter market efficiency across different time scales. Wang and Wang find significant scale heterogeneity in the entropy characteristics of equities, gold, Bitcoin, and the US dollar during the pandemic; Kuang characterizes information flows across international equity markets using a multi-scale entropy network; and Assaf et al. use Shannon and Rényi transfer entropy to distinguish directional linkages associated with common and tail events [39,40,41]. These findings suggest that a fixed window can confound short-run panic with medium- to long-term structural change, while the Rényi parameter can further alter a model’s sensitivity to different regions of the probability distribution.

Applications of entropy to sentiment, green assets, and energy markets provide further evidence of its nonlinear advantages. Caferra identifies regime-specific information transmission between sentiment and equity and cryptocurrency markets, while Dhifaoui et al. quantify cross-scale information flows among clean energy, carbon, crude oil, and precious metals [42,43]. Tong et al. find that tail observations contain information distinct from that at the center of the distribution, whereas Shternshis et al. emphasize that symbol partitioning and sampling frequency affect entropy estimates [44,45]. Entropy variables must therefore be defined with explicit windows, embedding dimensions, and bias-correction rules, rather than treated as assumption-free universal indicators.

The direction of information transmission is likewise time-varying around major events. Ferreira and Morais examine Rényi transfer entropy among green markets, structural shocks, and sentiment, while Jin and Xue characterize transmission networks between investor attention and stock-market behavior before and after the Russia–Ukraine conflict [46,47]. Methodologically, Chen et al. use ordinal-pattern transition networks to jointly exploit pattern distributions and transition probabilities, whereas Obanya et al. demonstrate the complementarity between permutation entropy and GARCH volatility series [48,49]. These studies show that distinct entropy measures capture different dimensions of complexity, probability concentration, and information direction and should therefore be integrated selectively.

Studies closer to the present setting document pronounced multi-scale changes in equity-market efficiency and global equity tail spillovers during crises [50,51]. Effective transfer entropy and quantile-on-quantile transfer entropy further reveal time variation and extreme-state dependence among oil-price uncertainty, climate-policy uncertainty, and new-energy markets [52,53]. Existing studies, however, mostly use entropy for ex post efficiency evaluation, spillover networks, or ordinary input variables; few allow it to directly govern the division of temporal-scale responsibilities within a VaR model.

Non-extensive entropy provides a related approach to market complexity. Tsallis [54] generalizes Boltzmann–Gibbs entropy through a deformation parameter. Loukeris and Gradojevic [55] incorporate Tsallis statistics and fractal market assumptions into the Evolving Returns Integrated System (ERIS), which evaluates modular, Jordan–Elman recurrent, and generalized feedforward neural models for portfolio selection. This connects non-Gaussian return behavior with neural modeling, whereas EntropyFusion-VaR uses WPE, Rényi entropy, and TE as state descriptors and an exponential log-sum-exp aggregation of quantile losses. The latter objective does not implement Tsallis entropy or introduce its deformation parameter.

Motivated by this gap, the present study specifies a causal dilated TCN as the local expert and a Patch Transformer as the long-memory expert, and feeds WPE, Rényi entropy, and transfer entropy into a dynamic gate so that market complexity and information flows directly determine the experts’ weights. This design addresses the inability of a fixed single backbone to adapt to multiple regimes [19,25,35] and advances entropy from an explanatory measure to a testable control signal [40,43,52]. In addition, an incremental cumulative mapping structurally ensures the monotonicity of the 1%, 2.5%, and 5% quantiles, thereby avoiding the distributional distortion caused by ex post sorting [27,29,30].

For evaluation, this study combines an entropic robust quantile loss, coverage calibration, standard backtests, and ablation experiments to address sparse extreme-tail observations, clustered exceptions, and model uncertainty [12,16,31]. The contribution thus lies not in merely stacking networks and entropy features, but in closing the loop of “entropy-based state identification–multi-scale expert specialization–non-crossing joint-quantiles–robust out-of-sample evaluation”. This framework jointly tests predictive accuracy, empirical coverage, structural consistency, and component-level interpretation.

## 3. Variable Definitions, Data Description, and Feature Engineering

Reliable extreme-risk forecasting depends not only on an advanced model architecture but also on high-quality inputs and substantive feature engineering. This section details the data sources, the logic used to partition the sample, and the construction of fundamental micro-level, macro-level, and risk-state variables. Particular attention is paid to the computation of multi-scale entropy features and their economic meaning as measures of financial-market complexity.

### 3.1. Sample Selection and Dataset Partitioning (Description of the Dataset A/B/C Splits)

The underlying daily equity-market data, including open, high, low, close, and volume, together with S&P 500 constituent-classification information, are drawn from the public Kaggle database “S&P 500 Stocks (daily updated)”. To evaluate performance across different segments of the energy value chain, three securities are selected to form separate test panels (Panel A/B/C): EOG Resources (EOG), an upstream oil-and-gas exploration and production company; Marathon Petroleum (MPC), a downstream refining and marketing company; and Schlumberger (SLB), an oilfield services and equipment company. To prevent future information from entering the training process and to ensure that model comparisons use identical temporal splits, the sample is divided into three non-overlapping subperiods: a training set covering 2016 through 2020 (including the high-volatility onset of the pandemic in 2020), a 2021 validation set used for early stopping and hyperparameter selection, and a test set extending from the beginning of 2022 through August 5, 2026 (used exclusively for out-of-sample evaluation). Detailed descriptions of the datasets and their trading-day counts are provided in Table 1.

### 3.2. Construction of Fundamental and Common Risk-State Variables

Efficient learning by deep models requires the feature-based representation of established financial priors. From the raw daily price and volume data, this study constructs a three-dimensional fundamental feature matrix covering microstructure and momentum, historical volatility and idiosyncratic risk, and common market conditions. For microstructure and momentum, the intraday high–low range and overnight price gap are extracted to characterize intraday trading disagreement and out-of-hours information shocks. For historical volatility, rolling realized volatility over multiple windows (5, 20, and 60 days) is constructed to measure volatility clustering. Downside semivariance and maximum interval drawdown are also introduced specifically to capture asymmetric market responses to declines and the current degree of selling pressure on each stock. To isolate systematic macro risk, return and volatility state features for the S&P 500 market and the equal-weighted energy sector are generated synchronously. The full construction and financial interpretation of these input-variable groups are summarized in Table 2.

### 3.3. Computation and Economic Meaning of Multi-Scale Entropy Features (WPE, Rényi, and TE)

One of the central innovations of this study is to distill multidimensional entropy theory into the principal signal source driving the deep model’s “entropy gate”. To comprehensively measure time-series complexity and patterns of information flow in energy markets, three categories of multi-scale entropy features are constructed. First, weighted permutation entropy (WPE) is calculated over 20-, 60-, and 120-day windows to further characterize the complexity of local ordinal patterns in returns and distinguish short-run disturbances from more persistent regime changes. Second, second-order Rényi entropy (q = 2) is used to characterize changes in the concentration of the return distribution and serves as a distributional-state measure complementary to WPE. Finally, transfer entropy (TE) from the market and the energy sector to each stock is estimated over a rolling window of 126 trading days. It measures the conditional information increment obtained by adding the history of an external series after controlling for the stock’s own history and is commonly used to describe nonlinear directional information flow. For these key targets and complex features, Table 3 reports detailed descriptive statistics for the full samples (encompassing the training, validation, and test sets) of all three datasets.

### 3.4. Time-Varying Evolution of Multi-Scale Entropy Features and Stylized Facts

Having defined the fundamental variables and presented full-sample descriptive statistics, this study next examines the dynamic evolution of the core entropy features during the test period. Because the efficiency of information transmission and the complexity of market microstructure differ substantially across macroeconomic regimes, static descriptive statistics cannot reveal shifts in market conditions immediately before the onset of extreme risk. This section therefore uses the time-series behavior of multi-scale weighted permutation entropy and transfer entropy to illustrate the complexity of the risk environment surrounding energy equities and to provide direct empirical motivation for the “dynamic gating” design introduced below.

**Figure 1:**
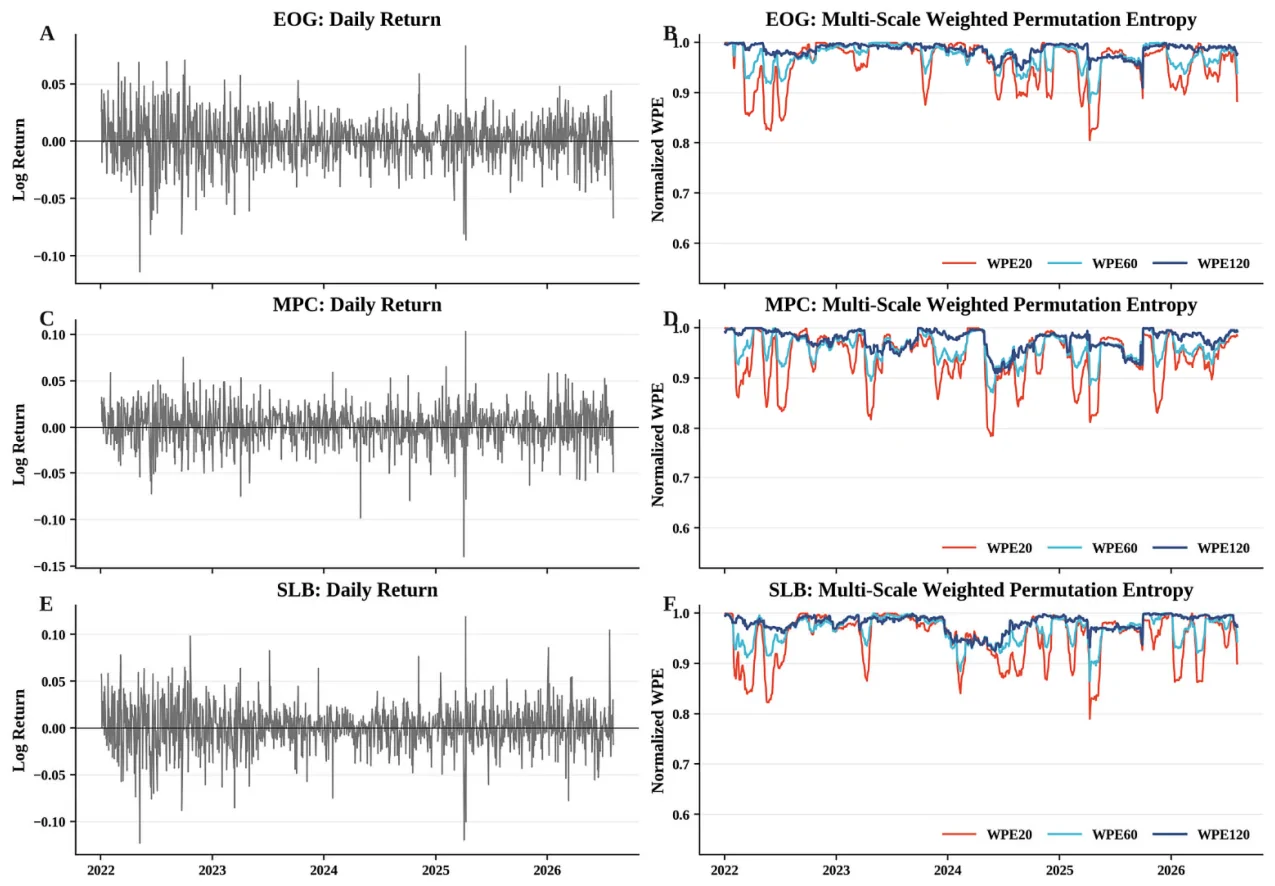
Time-series comparison of individual-stock returns and multi-scale weighted permutation entropy (WPE): (**A**) EOG returns; (**B**) EOG WPE; (**C**) MPC returns; (**D**) MPC WPE; (**E**) SLB returns; (**F**) SLB WPE.

We first examine the relationship between multi-scale WPE and fluctuations in individual-stock returns. The short-window WPE20 responds more rapidly to local price jumps, whereas WPE60 and WPE120 are smoother, reflecting the distinct ability of different scales to characterize short-run disturbances and persistent states. Episodic changes in WPE can be observed around certain pronounced downturns, suggesting that WPE is better understood as state information than as a deterministic leading indicator of extreme risk. Figure 1 directly compares the daily return series of EOG, MPC, and SLB with the evolution of their corresponding multi-scale WPE trajectories.

**Figure 2:**
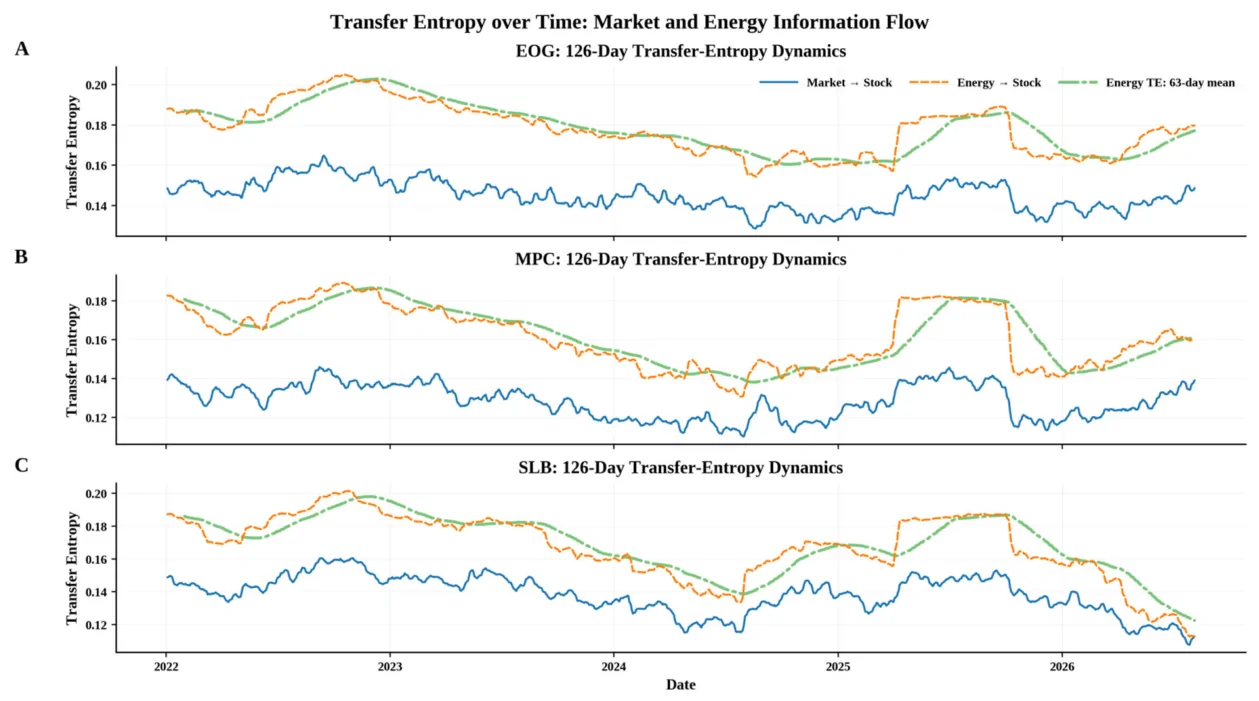
Time series of transfer entropy from the market and the energy sector to individual stocks: (**A**) EOG; (**B**) MPC; (**C**) SLB.

Second, this study examines dynamic information flows from the market and the energy sector to individual stocks. The strength of these flows is markedly time-varying and differs across stocks. Several stress episodes are accompanied by relatively high transfer entropy for different stocks, but the timing and duration of the peaks are not identical. This pattern suggests that external information flows may vary with market conditions and provides direct empirical motivation for the dynamic gate introduced below. Figure 2 presents the dynamic trajectories of transfer entropy from the market and the energy sector to each stock over rolling windows of 126 trading days in a dual-series time plot.

## 4. Core Methodology: EntropyFusion-VaR Model Design

To address the nonlinear time variation, quantile crossing, and distorted fitting of extreme outcomes in small samples that complicate extreme-tail-risk forecasting in energy equity markets, this study proposes a novel deep-learning architecture: the multi-scale entropy-gated deep quantile learning model (EntropyFusion-VaR). This section details the model’s core components, mathematical principles, and financial logic and clearly defines all notation and variables.

### 4.1. Overview of the Overall Model Architecture

The model’s primary objective is to use historical data to predict the set of extreme left-tail quantiles of future returns, that is, Value at Risk (VaR). The model input is *X_t_*_−*L*+1:*t*_, which denotes, at the current time t, a time-series matrix spanning L historical trading days (*L* = 120 in this study). The dimensions of this matrix are *L* × *d_in_*, where *d_in_* denotes the dimension of the fundamental feature matrix (e.g., returns, trading volume, and market volatility).

The forecast target is defined as the conditional left-tail quantile of the cumulative log return over the next *h* trading days. Specifically, the cumulative return is given by:

(1) $$y_{t,h}=\sum_{j=1}^{h}r_{t+j}=\ln(P_{t+h}/P_{t})$$

the forecast horizon *h* ∈ {1,5,10}. The model’s output target is:

(2) $$Q_{\alpha ,t}^{\left(h\right)}=Q_{\alpha }(y_{t,h}|F_{t})$$

The set of tail quantiles of interest is:

*α* ∈ {0.01,0.025,0.05} (3)

Forecasting is therefore not limited to the next day: *h* = 5 and *h* = 10 correspond to VaR for cumulative returns over the next 5 and 10 days, respectively.

**Figure 3:**
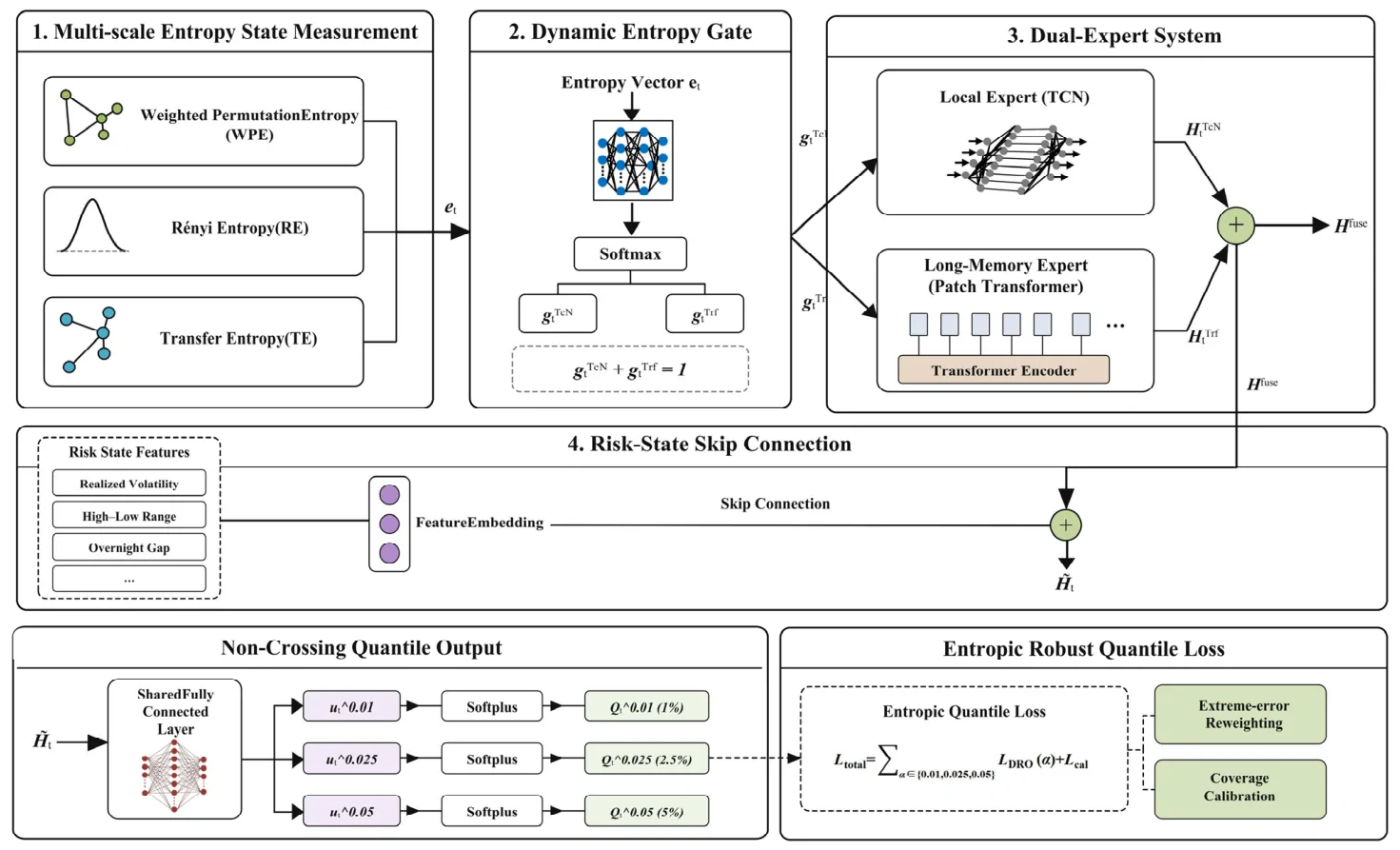
Overall network architecture of EntropyFusion-VaR. Rényi inputs describe concentration in the full binned return distribution.

The overall EntropyFusion-VaR architecture comprises four key modules. First, the multi-scale entropy-state encoder computes and encodes multidimensional information-theoretic measures, generating a state vector that represents the current degree of market “disorder”. Second, the dual-expert temporal feature-extraction branch uses a “causal dilated TCN (local expert)” to extract short-run nonlinear price-jump signals and a “Patch Transformer (long-memory expert)” to extract long-run macro-level memory dependencies. Third, the dynamic entropy gate and risk-state bypass use the entropy-state vector to allocate weights adaptively between the two experts and forcibly inject conventional financial risk factors through a direct residual path. Fourth, non-crossing quantile outputs and robust optimization feed the features into an output network with strict monotonicity and use a distributionally robust optimization mechanism to enhance sensitivity to extreme outcomes. The overall information flow and connections among these core modules are illustrated in Figure 3.

### 4.2. Multi-Scale Entropy-State Encoder (Entropy Encoder)

To capture the instantaneous complexity of financial markets, three key entropy measures are extracted, concatenated, and encoded to serve as the “brain” that guides path selection in the deep network.

First, weighted permutation entropy (WPE) measures the degree of “disorder” in the local ordering of an individual stock’s return series while accounting for the absolute magnitude of return fluctuations. Let m denote the embedding dimension and *τ* denote the time delay. The time delay *τ* is used to extract data points at specified intervals (e.g., *x_t_*,*x_t_*_+*τ*_,*x_t_*_+2*τ*_) to construct the ordinal patterns, meaning the right-hand side of the equation implicitly depends on *τ* through the formation of these patterns. Let *p_w_*(*π_i_*) denote the probability distribution, after variance weighting within the specified window, of the occurrence of the particular ordinal pattern *π_i_* [56]. It is calculated as follows:

(4) $$\mathrm{WP}E_{t}(m,\tau )=-\sum_{i=1}^{m!}p_{w}(\pi_{i})\log p_{w}(\pi_{i})$$

Second, Rényi entropy (q = 2) characterizes concentration in the full binned return distribution. If *P_t_* = {*p*_1,*t*_,*p*_2,*t*_,…,*p_n_*_,*t*_} denotes the empirical probability of observations falling into each interval within the historical rolling window, the corresponding entropy is defined by Equation (5).

For q = 2, squaring the bin probabilities gives greater weight to more populated bins. The entropy depends on these probabilities and does not identify whether a bin lies in the center or the left tail. We therefore interpret it as an overall distributional-state descriptor complementary to ordinal-pattern complexity and directional information flow. The No-Renyi ablation evaluates its incremental predictive value within the model.

(5) $$H_{2}(P_{t})=-\log\left(\sum_{i=1}^{n}p_{i,t}^{2}\right)$$

Finally, transfer entropy (TE) quantifies the lagged, nonlinear influence transmitted from the aggregate market or energy-sector state M to an individual stock S. With the stock’s own historical trajectory $s_{t}^{\left(k\right)}$ known, it measures how adding historical information from the aggregate market $m_{t}^{\left(l\right)}$ reduces predictive uncertainty about the stock’s future state *s_t_*_+1_:

(6) $$TE_{M\to S}(t)=\sum_{s_{t+1},s_{t}^{\left(k\right)},m_{t}^{\left(l\right)}}p(s_{t+1},s_{t}^{\left(k\right)},m_{t}^{\left(l\right)})\log\frac{p(s_{t+1}|s_{t}^{\left(k\right)},m_{t}^{\left(l\right)})}{p(s_{t+1}|s_{t}^{\left(k\right)})}$$

The encoder concatenates the features above and passes them through a multilayer feedforward network with a learnable weight matrix *W_enc_*, a trainable bias vector *b_enc_* (which is optimized during the neural network’s training phase via backpropagation to shift the activation threshold), and an activation function *σ*, outputting a low-dimensional “entropy hidden-state vector” that represents the current market-system environment $h_{t}^{\mathrm{entropy}}$:

(7) $$h_{t}^{\mathrm{entropy}}=\sigma \left(W_{\mathrm{enc}}[\mathrm{WP}E_{t}⊕H_{2,t}⊕TE_{t}]+b_{\mathrm{enc}}\right)$$

### 4.3. Dual-Expert Temporal Feature Extraction: Causal Dilated TCN and Patch Transformer

Because energy markets are sometimes dominated by short-run panic selling and at other times by long-run macroeconomic cycles, this study places two deep feature-extraction “experts” with different emphases in parallel.

Local Expert (TCN): The local expert uses causal dilated convolution [57] applied directly to the fundamental input features *X_t_*_−*L*+1:*t*_, rather than the entropy state $h_{t}^{\mathrm{entropy}}$. “Causality” ensures that the model strictly excludes future data, while “dilation” expands the receptive field by scanning the sequence at intervals. *X*^(*l*−1)^ denotes the output feature matrix from the preceding network layer. *f*(*i*) denotes the *i*-th learned weight parameter of the 1D convolution kernel of length K. The dilation rate *d* increases exponentially with the layer depth *l* (i.e., *d* = 2*^l^*). This specific exponential growth allows the network’s receptive field to expand rapidly, ensuring the effective capture of longer-term historical dependencies without requiring an excessively deep architecture or losing local resolution. The TCN responds rapidly to recent episodes of severe volatility, and the representation extracted at its final time step is denoted as the local risk vector $Z_{t}^{\mathrm{local}}$.

Long-memory expert (Patch Transformer): Similar to the TCN, the Patch Transformer [58] operates on the fundamental input features. To enable the self-attention mechanism to focus more effectively on long-horizon patterns, it is not applied to individual daily observations. Instead, the L-day input sequence is first divided into several equally sized, non-overlapping patches (i.e., “time segments”). Each patch is dimensionally reduced by the linear matrix *W_P_* and augmented with the positional encoding *E_pos_*, forming the patch-embedding vector *E_patch_*. Multi-head attention then computes inter-patch dependence weights to extract long-run trends:

(8) $$\mathrm{Attention}(Q,K,V)=\mathrm{Softmax}\left(\frac{QK^{T}}{\sqrt{d_{k}}}\right)V$$

Here, *Q* (query), *K* (key), and *V* (value) are not derived from $h_{t}^{\mathrm{entropy}}$, but are obtained through the matrix multiplication of the patch-embedding vector *E_patch_* with their respective learned projection matrices (i.e., *Q* = *E_patch_W_Q_*, *K* = *E_patch_W_K_*, *V* = *E_patch_W_V_*). *d_k_* denotes the dimension of the key vectors, and $\sqrt{d_{k}}$ serves as a scaling factor to prevent the dot products from growing excessively large, which would otherwise push the Softmax function into regions with extremely small gradients. Finally, Softmax is the normalized exponential function that converts the scaled dot products into a probability distribution of attention weights that sum to 1. This branch effectively extracts long-memory dependencies spanning monthly horizons, and its output feature vector is denoted by $Z_{t}^{\mathrm{long}}$.

### 4.4. Dynamic Entropy Gate and Risk-State Skip

Dynamic Entropy Gate: Rather than simply averaging the two experts, the model feeds the “entropy hidden state” $h_{t}^{\mathrm{entropy}}$ into the gating network, where it is mapped by *W_g_* (the learnable weight matrix of the gating network that projects the state vector into a 2-dimensional space corresponding to the two experts) and then passed through a Softmax activation function. This operation outputs two normalized probability weights between 0 and 1 that sum to 1: $w_{t}^{\mathrm{local}}$ (assigned to the TCN) and $w_{t}^{\mathrm{long}}$ (assigned to the Transformer):

(9) $$\left[\begin{array}{c} w_{t}^{\mathrm{local}}\\ w_{t}^{\mathrm{long}} \end{array}\right]=\mathrm{Softmax}(W_{g}\cdot h_{t}^{\mathrm{entropy}}+b_{g})$$

Note that while the Softmax mathematical operation here is identical to the one used in Equation (8), it is applied across the 2-dimensional expert space to ensure the generated fusion weights sum exactly to 1. In this context, b_g_ is the trainable bias vector of the gating network, which functions to adjust the baseline selection preference for either expert when the entropy state is neutral. The gating network does not impose a fixed monotonic relationship between any state variable and either the local or long-memory expert. Instead, the two experts’ weights are jointly determined by multidimensional entropy states and adjusted in both directions across market regimes. Dynamic feature fusion is completed by their weighted sum:

(10) $$Z_{t}^{\mathrm{fused}}=w_{t}^{\mathrm{local}}\cdot Z_{t}^{\mathrm{local}}+w_{t}^{\mathrm{long}}\cdot Z_{t}^{\mathrm{long}}$$

Risk-State Skip: During forward propagation, a deep network may excessively abstract or even lose conventional financial measures that are intuitively effective (e.g., deterministic features such as the maximum drawdown over the previous 60 days and annualized volatility $X_{t}^{\mathrm{risk}}$). This study therefore designs a shortcut skip path. Wskip is the weight matrix that directly maps the fundamental features, and *b_skip_* is the corresponding trainable bias vector. This bias vector adjusts the numerical baseline of the projected risk features so that they appropriately align with the deep fused features before the addition operation. Next, the original risk features are added directly to the fused features $Z_{t}^{\mathrm{fused}}$, and LayerNorm removes scale differences, producing the complete final hidden vector $H_{t}^{\mathrm{final}}$:

(11) $$H_{t}^{\mathrm{final}}=\mathrm{LayerNorm}(Z_{t}^{\mathrm{fused}}+W_{\mathrm{skip}}X_{t}^{\mathrm{risk}}+b_{\mathrm{skip}})$$

The out-of-sample predictive contributions of the different risk features retained by the Risk-State Skip are examined further in Section 5.5.

### 4.5. Non-Crossing Multi-Quantile Output Layer

When forecasting left-tail loss VaR, basic financial logic requires low-probability extreme losses to have a greater magnitude than higher-probability ordinary losses; that is, the following condition must hold: ${\hat{q}}_{0.01}(t)<{\hat{q}}_{0.025}(t)<{\hat{q}}_{0.05}(t)$. When a conventional neural network outputs these three points independently, the corresponding curves can readily cross and intertwine.

To prevent this logical inconsistency at the architectural level, this study adopts an incremental cumulative output structure.

First, the final-state vector $H_{t}^{\mathrm{final}}$ and the weight matrix $W_{0.01}^{T}$ are used in a conventional linear mapping to directly predict the most extreme baseline point (1% VaR):

(12) $${\hat{q}}_{0.01}(t)=W_{0.01}^{T}H_{t}^{\mathrm{final}}+b_{0.01}$$

For the remaining, less extreme 2.5% and 5% forecast points, the model no longer directly predicts their absolute values. Instead, it uses the monotonic and strictly positive Softplus function (which satisfies Softplus(*x*) = log(1 + *e^x^*) > 0) to predict their “reverse increments” relative to the preceding, more extreme point:

(13) $${\hat{q}}_{0.025}(t)={\hat{q}}_{0.01}(t)+\mathrm{Softplus}(W_{0.025}^{T}H_{t}^{\mathrm{final}}+b_{0.025})$$

(14) $${\hat{q}}_{0.05}(t)={\hat{q}}_{0.025}(t)+\mathrm{Softplus}(W_{0.05}^{T}H_{t}^{\mathrm{final}}+b_{0.05})$$

Because the increments produced by Softplus are nonnegative, this parameterization guarantees *Q*0.01 ≤ *Q*0.025 ≤ *Q*0.05 and eliminates the need for ex post manual sorting.

The out-of-sample monotonicity and quantile-crossing rate of this non-crossing structure are examined further in Section 5.4.

### 4.6. Objective-Function Design: Entropic Robust Quantile-Loss and Coverage Calibration

Accurate quantile prediction by a deep model requires a specialized error function, namely pinball loss:

(15) $$L_{\alpha }(y_{t},{\hat{q}}_{\alpha }(t))=\max(\alpha (y_{t}-{\hat{q}}_{\alpha }(t)),(1-\alpha )({\hat{q}}_{\alpha }(t)-y_{t}))$$

*y_t_* denotes the observed return, and ${\hat{q}}_{\alpha }(t)$ denotes the specified quantile predicted by the model. Through the parameter *α*, this function assigns different penalty weights to “risk overestimation” and “risk underestimation”.

When estimating the 1% conditional quantile (i.e., *α* = 0.01), a mean loss can underemphasize a small number of observations with particularly large quantile forecast errors. Inspired by distributionally robust optimization (DRO), this study therefore uses an entropic robust quantile-loss function:

(16) $$L_{\mathrm{DRO}}(\alpha )=\frac{1}{\gamma }\log\left(\frac{1}{N}\sum_{i=1}^{N}\exp\left(\gamma \cdot L_{\alpha }(y_{i},{\hat{q}}_{\alpha }(i))\right)\right)$$

N is the number of observations in the training batch, and exp(⋅) is the natural exponential function. γ > 0 is the risk-sensitivity parameter. For a fixed training batch, differentiating Equation (16) with respect to each observation’s quantile loss yields positive weights proportional to its exponentiated, risk-scaled loss; these weights sum to one. Increasing *γ* therefore concentrates the optimization weight on observations with larger quantile losses. These can include severe downside forecast errors, but their weights depend on forecast loss rather than rarity alone. As the parameter approaches zero, Equation (16) approaches the mean pinball loss. Equation (17) separately regularizes a differentiable proxy for empirical exception frequencies. Neither exponential weighting nor this marginal-frequency penalty guarantees nominal conditional coverage.

Equation (16) defines an entropic risk-sensitive forecast-loss functional. Its minimizer targets exponentially weighted forecast loss rather than automatically retaining the ordinary expected-pinball quantile target. Under continuity and existence of the relevant exponential moment, an interior scalar minimizer satisfies the weighted score condition below. The expression characterizes the entropic component; the final objective in Equation (18) also includes coverage regularization.

E(exp(*γ*ℒ*_α_*(*Y*,*q_α_*))(I(*Y* < *q_α_*) − *α*)) = 0

This condition corresponds to an α-quantile under a loss-dependent exponential tilt, rather than under the original return distribution. As γ tends to zero, the entropic term approaches mean pinball loss. Large losses can arise from both downside misses and conservative risk forecasts, so exponential weighting emphasizes forecast errors rather than crash observations alone. The risk-sensitivity coefficient γ in Equation (16) is distinct from the WPE embedding delay used in Equation (4).

Coverage regularization is applied during training through a smooth exception-frequency proxy. The revised expression distinguishes this differentiable optimization term from the hard exceptions used for out-of-sample backtesting:

(17) $$L_{\mathrm{cal}}=\lambda \sum_{\alpha \in A}{\left({\hat{c}}_{\alpha ,\delta }-\alpha \right)}^{2}\;{\hat{c}}_{\alpha ,\delta }=\frac{1}{N}\sum_{i=1}^{N}\sigma \left(\frac{q_{\alpha }\left(i\right)-y_{i}}{\delta }\right)\;\sigma \left(z\right)=\frac{1}{1+\exp\left(-z\right)}$$

I(⋅) is retained for evaluation: it equals 1 when the observed return falls below the predicted quantile. The training expression in Equation (17) replaces this step function with a sigmoid proxy.

Here, σ is the logistic sigmoid, δ > 0 is its smoothing bandwidth, and λ is the calibration weight. The smooth frequency is differentiable with respect to the predicted quantiles; as δ tends to zero, it approaches the hard exception frequency away from ties. The penalty encourages the batch-level proxy to approach each nominal probability α. Realized exception rates and all coverage backtests continue to use hard indicators. Their agreement with the nominal probabilities is an empirical assessment separate from the training regularizer.

The final objective sums the three entropic quantile-loss terms and the smooth coverage regularizer. Quantile ordering follows from the output architecture, while coverage and predictive accuracy are assessed through separate out-of-sample diagnostics:

(18) $$L_{\mathrm{total}}=\sum_{\alpha \in \{0.01,0.025,0.05\}}L_{\mathrm{DRO}}(\alpha )+L_{\mathrm{cal}}$$

## 5. Experimental Results and Analysis—Core Benchmark Comparison

This section reports the main experimental results for EntropyFusion-VaR in sequence, including the benchmark models and experimental settings, forecasts of 1% extreme-tail risk, performance across stocks and quantiles, tests of the non-crossing constraint, and an interpretive analysis of the model’s internal mechanisms.

### 5.1. Benchmark Models, Experimental Settings, and Evaluation Framework

To comprehensively assess the effectiveness of EntropyFusion-VaR in forecasting extreme-tail risk, this study constructs a benchmark system spanning traditional econometric, machine-learning, and deep-learning approaches. The traditional econometric benchmarks are Historical Simulation (HS-500), EWMA-Normal, GJR-GARCH-t, and CAViaR-AS. The machine-learning benchmarks are QRF and Q-LightGBM, while the deep quantile benchmarks comprise LSTM-QR, TCN-QR, and Patch Transformer-QR. In addition, a fixed dual-expert fusion model (TCN+Patch-Fixed) is specified as a structural control to identify the incremental value of dynamic entropy gating relative to static weights.

Forecast performance is evaluated through a multidimensional backtesting framework. Predictive accuracy is measured primarily by quantile loss (pinball loss), while the exception ratio, coverage error, and exceedance severity characterize, respectively, the frequency with which realized returns fall below VaR, the calibration deviation from the theoretical quantile, and the magnitude of exceptions. Statistical validity under extreme confidence levels is evaluated jointly using the Kupiec unconditional coverage test (POF), the Christoffersen independence test (IND) and conditional coverage test (CC), and the dynamic-quantile (DQ) test. For the joint-quantile forecasting task, the quantile-crossing rate is also reported to determine whether the model’s outputs strictly follow the monotonic ordering required by financial logic. Cross-stock means describe the studied sample. In particular, an average of backtest *p*-values is not a pooled hypothesis test; statistical acceptance or rejection belongs to each stock-specific sequence.

The 1% tail requires particular attention to sampling uncertainty. With 1151 test observations per stock, nominal 1% coverage corresponds to only 11.51 expected exceptions. Under an independent Bernoulli reference model, the exception-rate standard error is about 0.293 percentage points, and the central 95% range is approximately 5–19 exceptions. These are reference quantities under nominal coverage, not confidence intervals calculated from the observed counts. Serially dependent exceptions can further change uncertainty. Reported loss gaps and stock–horizon rankings are therefore treated as descriptive comparisons unless a matched stock-specific significance test is available.

For the experimental design, all models are evaluated using exactly the same training, validation, and test-set partitioning rules. The deep-learning models share a historical input window of 120 trading days. The validation set is used only for early stopping and hyperparameter selection, whereas the test set is reserved exclusively for final independent out-of-sample evaluation. This design strictly prevents information leakage and look-ahead bias in model comparisons. The specific hyperparameter search spaces, network configurations, and optimal training settings for all benchmark models and EntropyFusion-VaR are provided in Appendix A, Table A1.

### 5.2. Out-of-Sample Forecasting and Backtesting Results for 1% Extreme-Tail VaR

**Figure 4:**
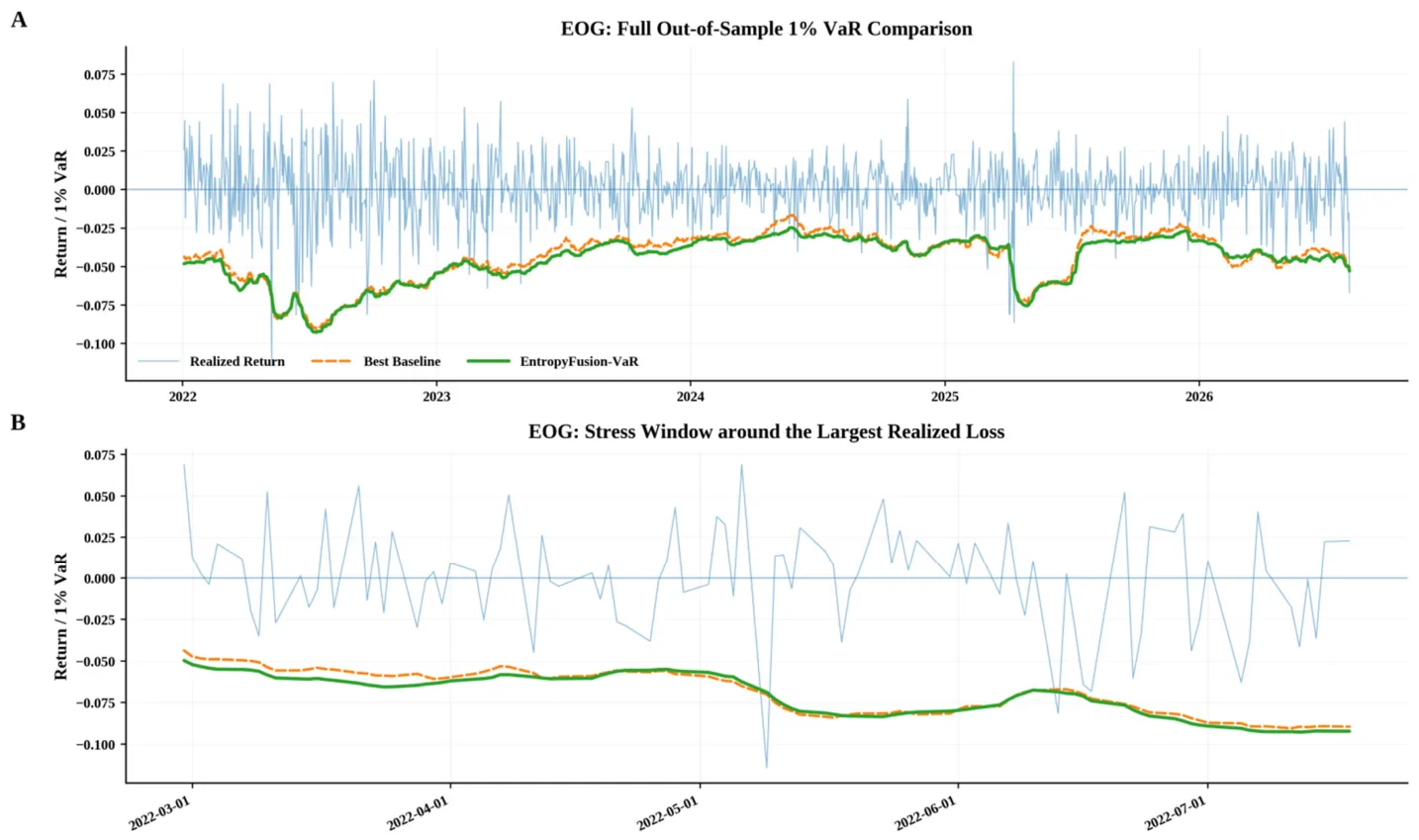
Out-of-sample 1% VaR forecasts and realized returns for EOG: (**A**) full test-period comparison; (**B**) stress-window comparison around the largest realized loss.

Table 4 reports cross-stock average forecasting and backtesting statistics for 1% VaR. EntropyFusion-VaR has an average pinball loss of 0.716 (×10^−3^), compared with 0.754 (×10^−3^) for the fixed dual-expert model, and an average exception rate of 1.01%. These descriptive averages summarize the three selected stocks; statistical coverage conclusions belong to the individual stock-specific backtests. Figure 4 illustrates forecasting behavior for EOG. Panels A and B show the full test-period trajectory and a stress window around the largest realized loss, respectively, allowing the adaptation of the forecast risk boundary to be inspected alongside realized returns.

### 5.3. Comparisons Across the Three Stocks and Multiple Quantiles

**Figure 5:**
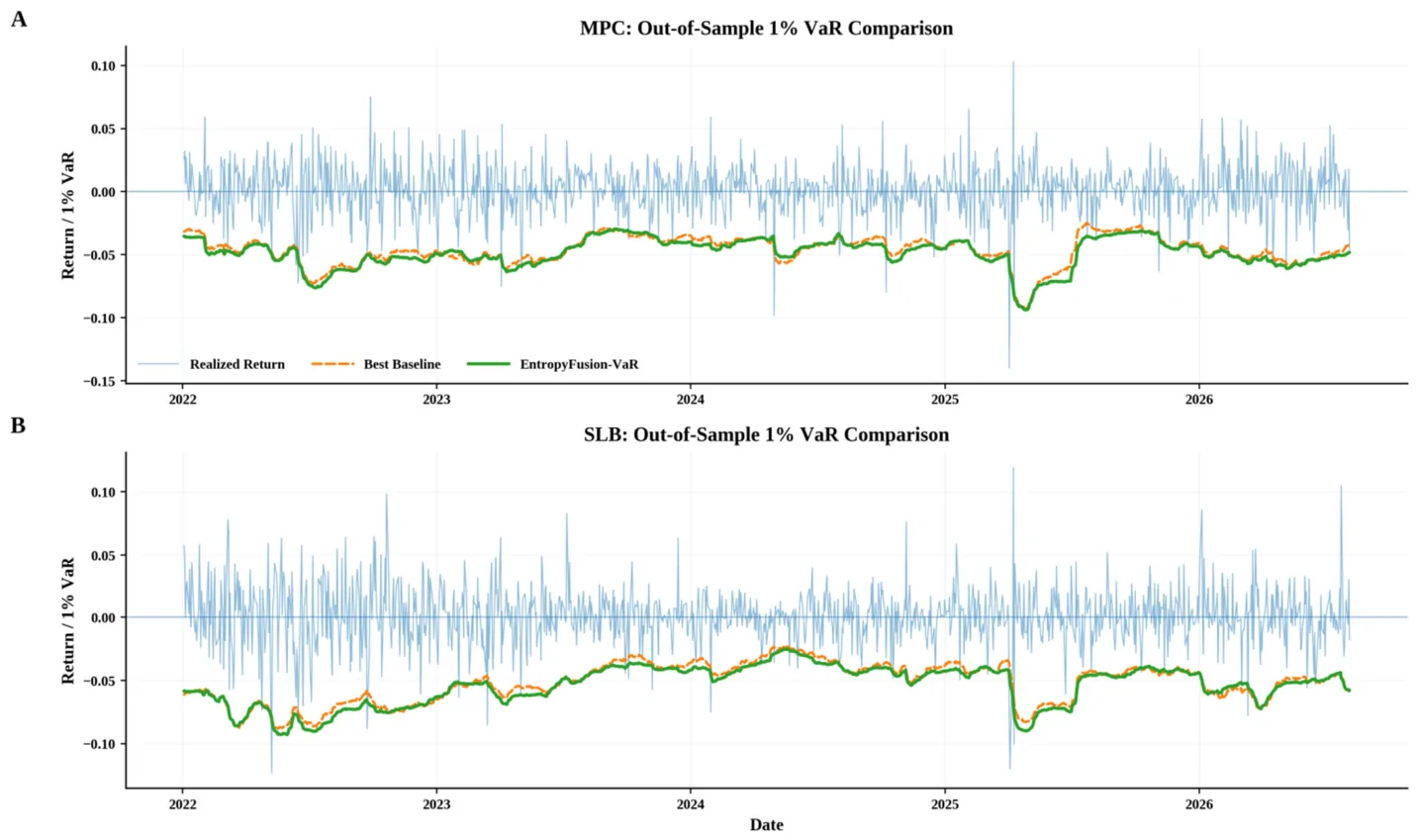
Comparison of out-of-sample 1% VaR forecasts and realized returns: (**A**) MPC; (**B**) SLB.

**Figure A1:**
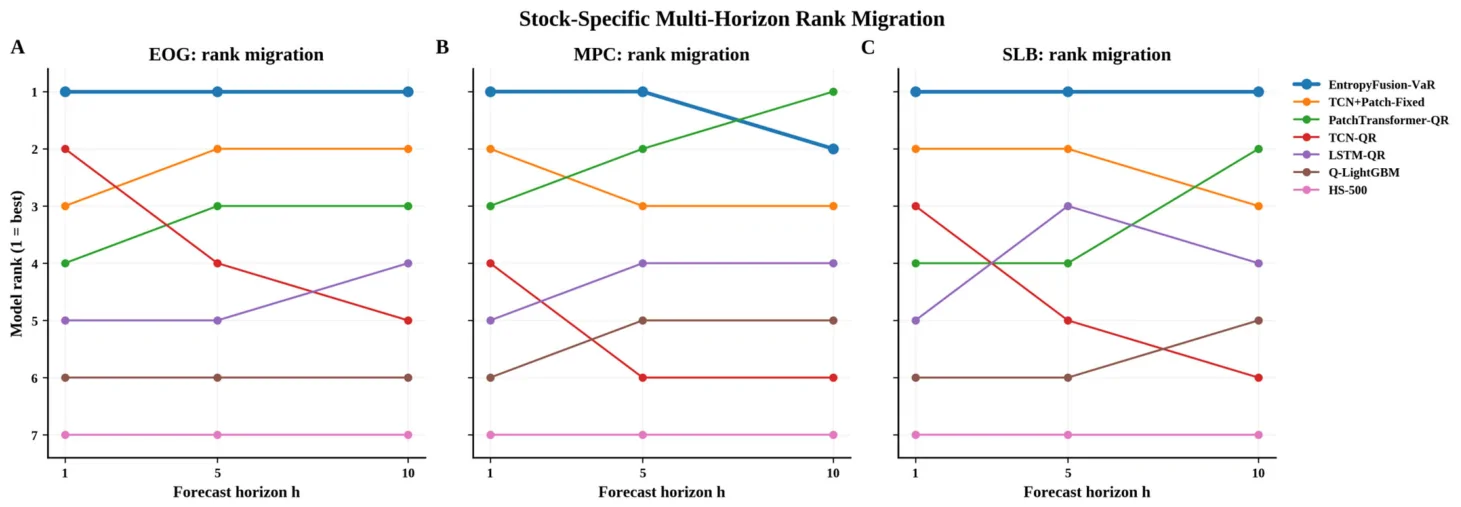
Stock-specific multi-horizon model rank migration: (**A**) EOG; (**B**) MPC; (**C**) SLB.

The comparison is extended to the 2.5% and 5% quantiles for the same three stocks. Table 5 reports the manuscript’s cross-stock average losses and backtesting summaries. EntropyFusion-VaR retains the lowest reported average loss of 1.113 (×10^−3^) at *Q*0.025; Patch Transformer-QR ranks second at *Q*0.05, showing that benchmark ordering varies with the target probability. These comparisons assess heterogeneity within EOG, MPC, and SLB. Figure 5 displays the 1% VaR paths for MPC and SLB and illustrates how risk forecasts adapt to the return dynamics of the downstream-refining and oilfield-services stocks. Multi-horizon model rankings are shown separately in Figure A1.

### 5.4. Effectiveness of Non-Crossing Joint-Quantile Outputs

**Figure 6:**
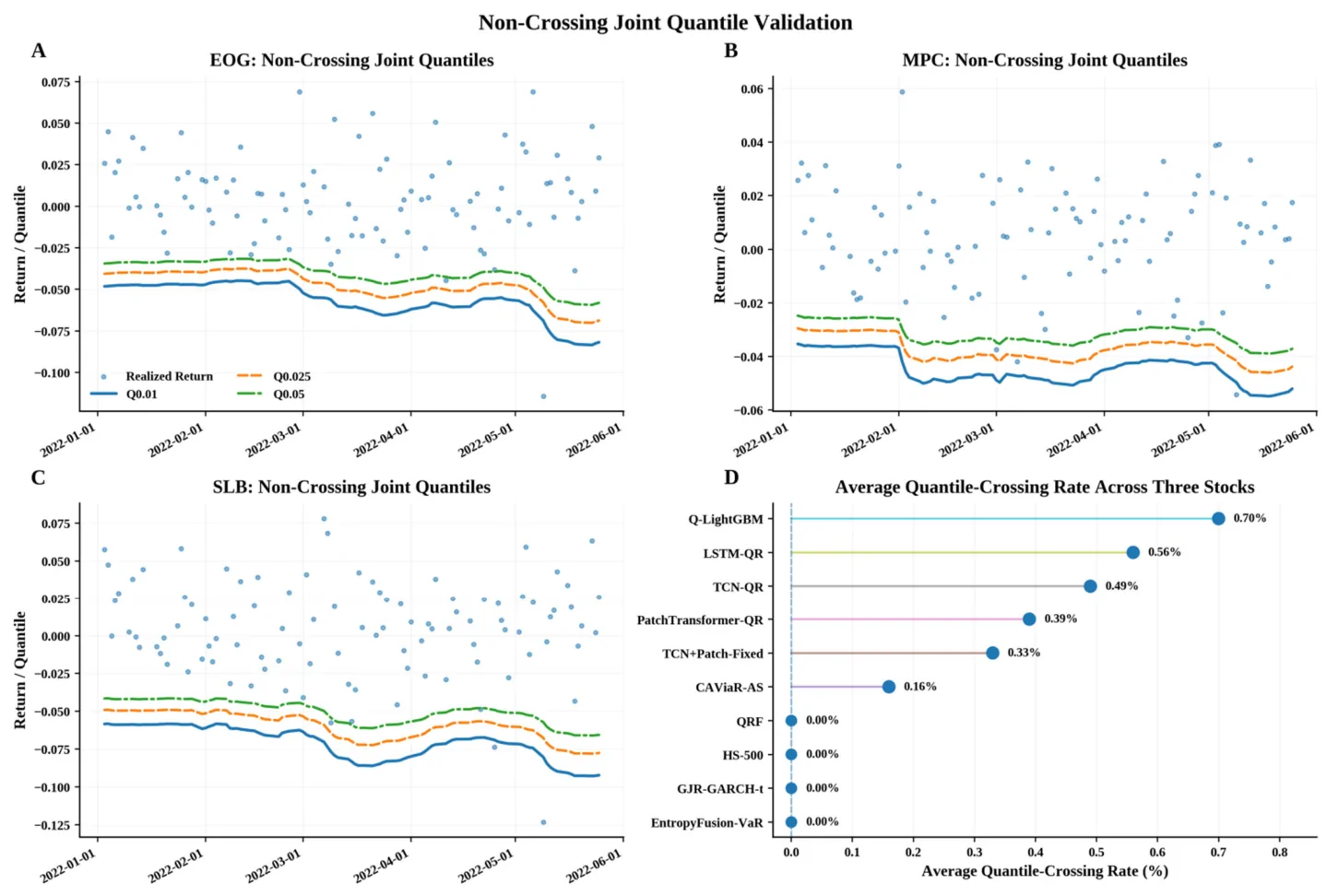
Validation of non-crossing joint-quantile outputs. Panels (**A**–**C**) show stock-specific paths. Panel (**D**) reports the cross-stock average crossing rates.

Joint-quantile forecasts should preserve their ordering across risk levels. The incremental cumulative output structure guarantees ordered quantiles for EntropyFusion-VaR. Figure 6A–C illustrates this ordering in the three stock-specific forecast paths, while Figure 6D reports average crossing rates. The displayed rates are 0.00% for EntropyFusion-VaR, 0.33% for TCN+Patch-Fixed, 0.39% for Patch Transformer-QR, 0.49% for TCN-QR, and 0.56% for LSTM-QR. Q-LightGBM and CAViaR-AS have displayed rates of 0.70% and 0.16%, respectively. The structural guarantee for the proposed output layer concerns consistency among the predicted quantiles; it does not by itself imply correct coverage.

### 5.5. Feature Contributions and Economic Interpretation of the Risk-State Skip

**Figure 7:**
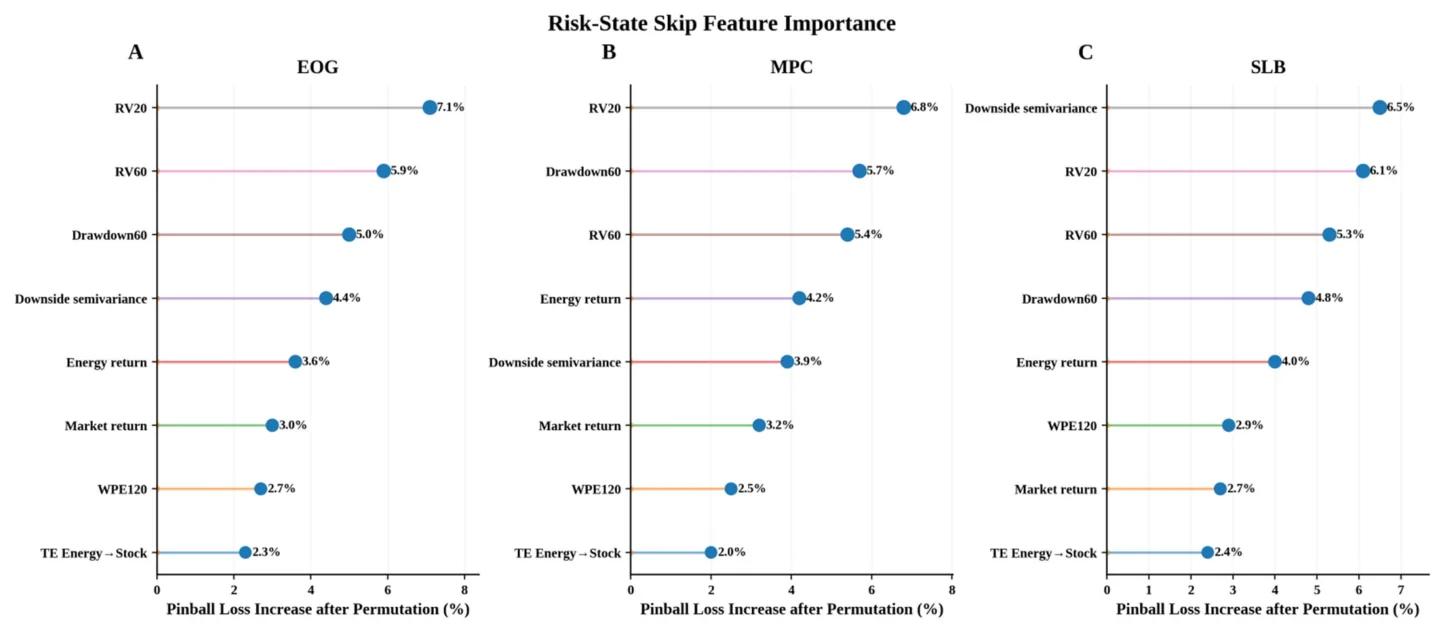
Visualization of feature importance for the Risk-State Skip: (**A**) EOG; (**B**) MPC; (**C**) SLB.

To identify the predictive information actually retained by the Risk-State Skip, permutation importance is used to quantify each feature’s contribution to model performance. Figure 7 combines horizontal lollipop plots with scatter points to visualize these importance estimates. In each of the three panels of Figure 7, the horizontal axis precisely measures the percentage increase in pinball loss caused by randomly permuting a given feature, and the features are ordered from top to bottom by the resulting increase in loss. The figure reveals clear heterogeneity in feature contributions. For example, 20-day and 60-day rolling volatility contribute the most in the EOG panel, whereas downside semivariance ranks first in the SLB panel. These shifts in the visual rankings not only demonstrate that conventional micro-level risk measures remain dominant contributors to prediction in deeper network layers but are also highly consistent with actual differences in risk exposure across segments of the energy value chain, strongly supporting the theoretical rationale for retaining conventional risk variables through a skip structure.

### 5.6. Dynamic Weights and Market-State Dependence of the Entropy Gate

**Figure 8:**
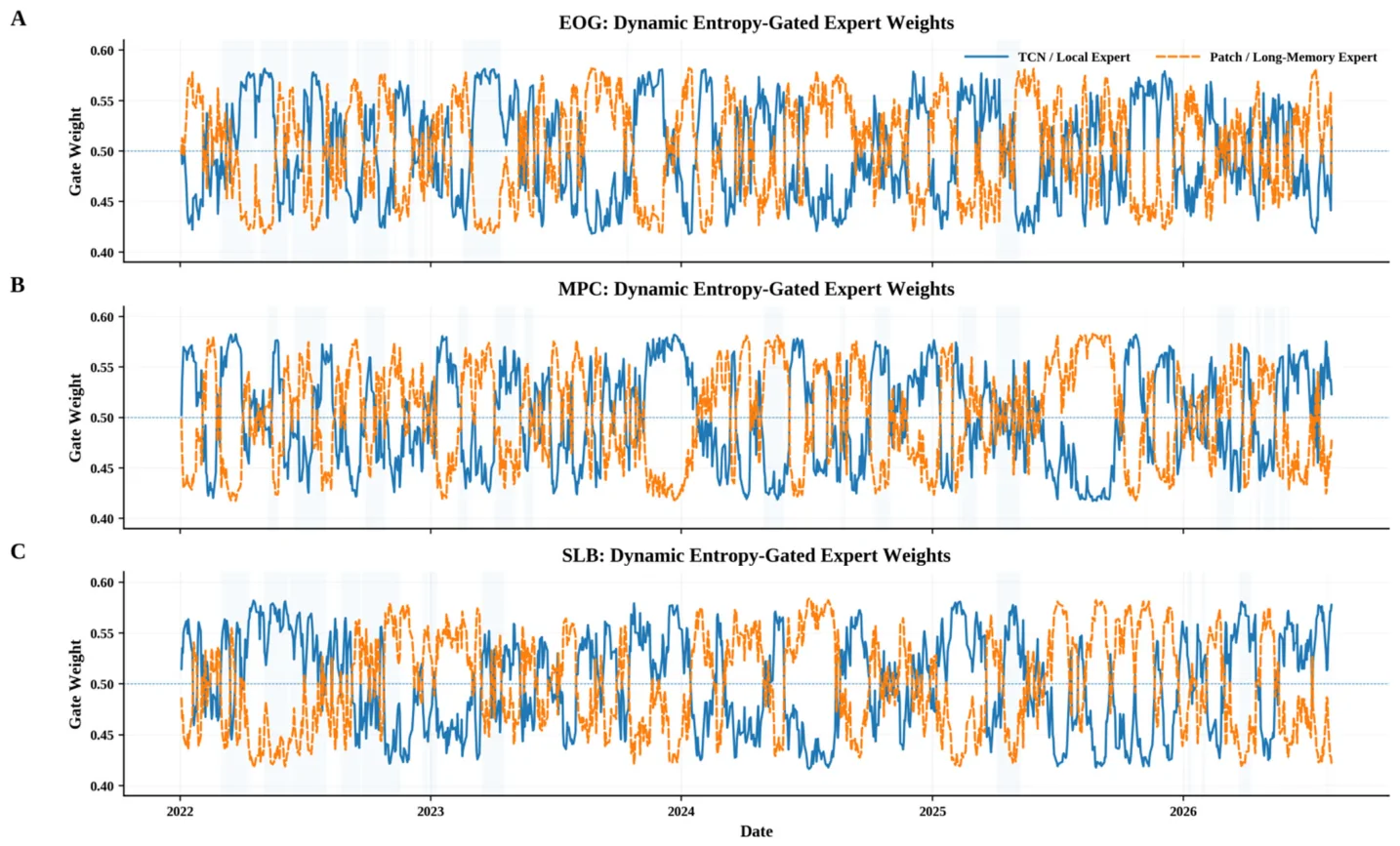
Time series of the entropy gate’s dynamic weight-allocation mechanism: (**A**) EOG; (**B**) MPC; (**C**) SLB.

This section investigates how the model’s core entropy-gating module adaptively allocates expert weights as market conditions evolve. Table 6 systematically reports Spearman correlations between the local-expert gating weight and multidimensional external market-state indicators. The results show that these state dependencies are generally weak to moderate and exhibit pronounced stock-level heterogeneity. To illuminate the mechanism underlying this dynamic allocation, Figure 8 uses intertwined dual-line time plots to trace the evolution of gating weights for all three stocks throughout the test period. The two curves representing the TCN local expert and the Patch Transformer long-memory expert display frequent and pronounced mirror-image fluctuations around the 0.5 reference line: at times the local expert dominates, while at others the allocation shifts rapidly toward the long-memory expert. This variation makes the allocation between the two experts observable, but it does not by itself identify the financial causes of a forecast or explain all hidden representations.

Interpretation therefore rests on complementary diagnostics. Permutation importance in Figure 7 quantifies predictive sensitivity to the Risk-State Skip inputs; gate paths and correlations in Figure 8 and Table 6 describe state-dependent allocation; and the component ablations in Table 7 assess changes in forecast loss. Together, these diagnostics support an interpretation at the feature and module levels. Permutation importance can be affected by dependence among inputs, and the observed gate correlations and ablation effects do not establish causal relationships.

## 6. Robustness Tests and Ablation Studies

### 6.1. Ablation Experiments on Core Components

**Figure A2:**
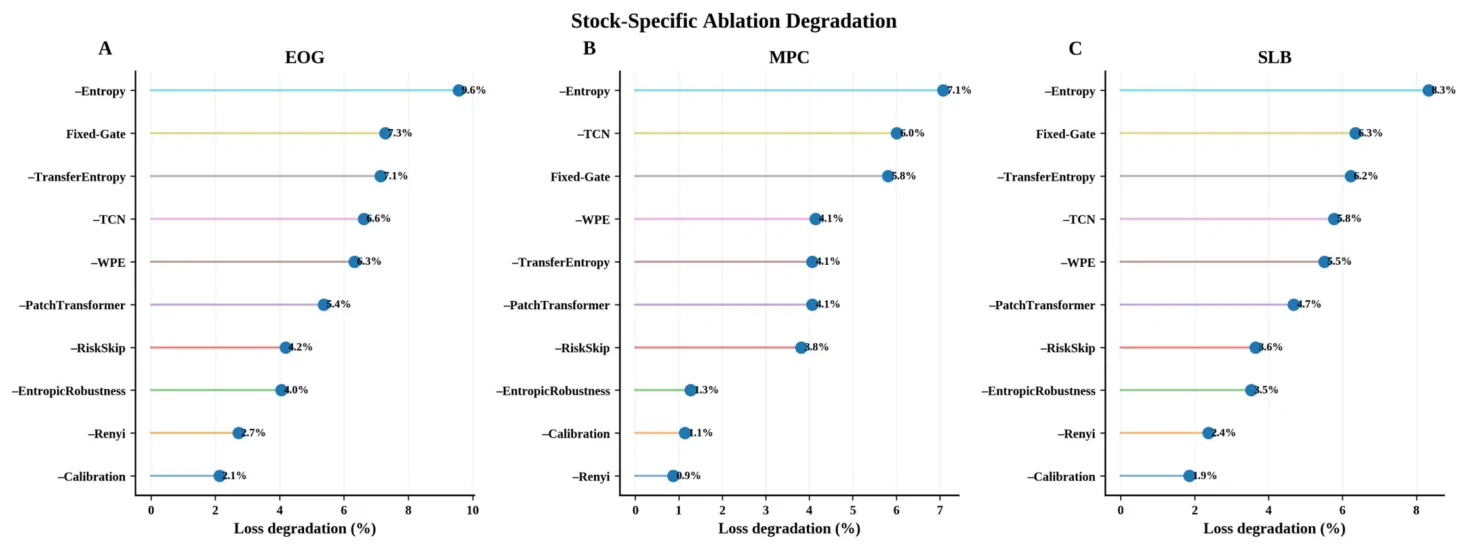
Stock-specific ablation degradation: (**A**) EOG; (**B**) MPC; (**C**) SLB.

Table 7 reports the stock-specific relative loss deterioration displayed in Figure A2 and the arithmetic mean of those displayed percentages. Removing all entropy inputs increases loss by 9.6%, 7.1%, and 8.3% for EOG, MPC, and SLB, respectively, giving a displayed-value mean of about 8.3%. Fixed gating, removal of the TCN, and removal of transfer entropy have mean deterioration of about 6.5%, 6.1%, and 5.8%, respectively. Removing calibration increases loss by 2.1%, 1.1%, and 1.9%, with a mean of about 1.7%. These are relative predictive-loss effects for the three stocks. They support incremental contributions from the evaluated modules, with the largest displayed mean deterioration following removal of all entropy features. The calibration ablation concerns loss and is not, by itself, evidence of a statistically significant coverage improvement.

### 6.2. Parameter Sensitivity and Distributional Robustness of the Loss Function

**Figure 9:**
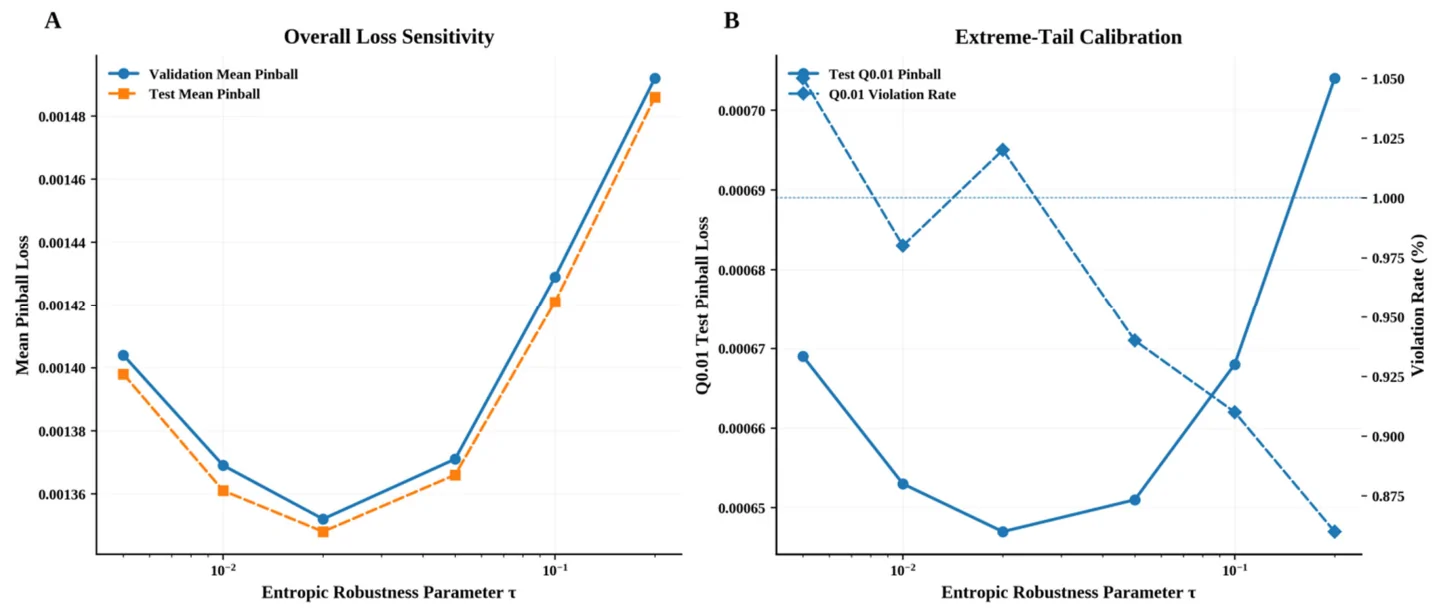
Line plot of tail-loss sensitivity to the entropic robustness parameter τ: (**A**) overall loss sensitivity, showing validation and test mean pinball loss; (**B**) extreme-tail calibration, showing test Q0.01 pinball loss and Q0.01 violation rate.

**Figure 10:**
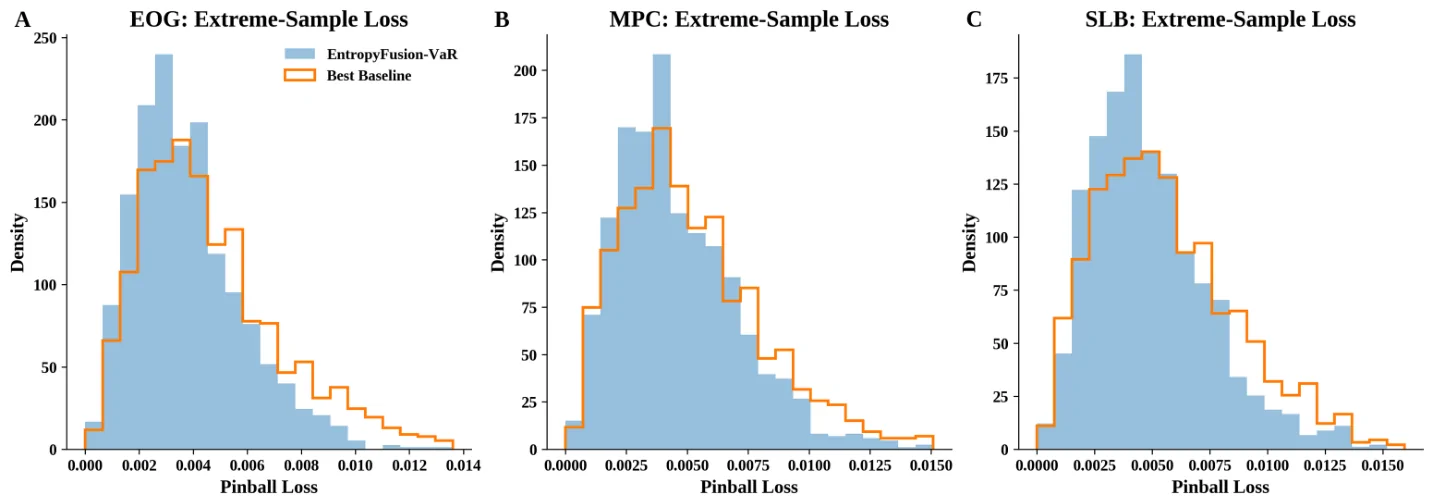
Histogram of model pinball loss at extreme observations: (**A**) EOG; (**B**) MPC; (**C**) SLB.

Figure 9 describes sensitivity to the risk-sensitivity coefficient, denoted τ in the figure and γ in Equation (16). The validation- and test-loss curves are approximately U-shaped, with relatively low values near 0.02. The right-hand panel shows the accompanying changes in the 1% loss and exception rate. These curves provide a sensitivity diagnostic rather than a new absolute-loss benchmark for Table 4 and Table 5. Table 8 now isolates the loss-objective variants using the relative loss changes displayed in Figure A2: removing the entropic component while retaining calibration, removing calibration while retaining the entropic component, and retaining both terms. This comparison uses the same relative reporting convention as Table 7. Figure 10 complements the comparison by showing the plotted loss distributions for EntropyFusion-VaR and the best baseline; the right-tail shapes describe the occurrence of large forecast errors in those displays.

Forecast-loss robustness and quantile coverage are assessed separately. Table 8 reports relative loss deterioration, whereas exception rates and coverage backtests are reported in Table 4 and Table 5. The 1%, 2.5%, and 5% VaR levels are nominal tail probabilities. A displayed loss distribution or a reduction in large forecast errors is not a coverage test. Figure 9 supports a sample-specific sensitivity description and does not identify a universally optimal risk-sensitivity coefficient. The plotted τ is the risk-sensitivity coefficient γ in Equation (16), distinct from the WPE embedding delay.

### 6.3. Multi-Horizon Validation

Table 9 summarizes the rankings of the seven models displayed in Figure A1 over horizons of 1, 5, and 10 trading days. EntropyFusion-VaR ranks first in eight of the nine stock–horizon combinations and has the lowest cross-stock mean rank at each horizon: 1.00, 1.00, and 1.33. It ranks first at all horizons for EOG and SLB and at the 1- and 5-day horizons for MPC. The exception is the MPC 10-day forecast, where it ranks second behind Patch Transformer-QR. Patch Transformer-QR’s mean rank improves from 3.67 at one day to 2.00 at ten days, whereas TCN-QR’s mean rank changes from 3.00 to 5.67. These results document horizon-dependent ordering in this sample; mean rank and absolute pinball loss are distinct summaries.

The multi-day targets are cumulative log returns as defined in Equation (1). When such return windows overlap, shared daily returns can induce dependence between forecast targets. Table 9 and Figure A1 are therefore interpreted as descriptive comparisons of horizon-specific forecasting performance, rather than significance tests that treat multi-day observations as independent.

### 6.4. Analysis of Model Efficiency for Small Samples of Individual Stocks

In real-world financial-engineering applications, strong predictive accuracy must be matched by reasonable computational cost to meet industrial requirements. Table 10 objectively evaluates the computational efficiency of EntropyFusion-VaR and the benchmark models along three core dimensions: the total number of trainable network parameters, fitting time per dataset, and single-step inference latency. The results in Table 10 show that EntropyFusion-VaR contains approximately 8.27 × 10^4^ trainable parameters, resulting in a complete fitting time of approximately 5.65 s per dataset and a single-observation inference latency of 0.105 milliseconds. Its absolute computational cost is therefore higher than that of very parsimonious traditional models such as GJR-GARCH-t. With modern computing resources, however, this microsecond-scale inference latency readily satisfies the practical requirement of daily risk-boundary updates in financial institutions. More importantly, EntropyFusion-VaR exchanges a moderate increase in hardware computation for a substantial improvement in the accuracy of extreme-tail forecasts. Overall, the model overcomes the limitations of traditional models in fitting nonlinearity and extremes while maintaining high response efficiency. It achieves a reasonable balance between gains in accuracy and computational cost and thus exhibits favorable engineering cost-effectiveness and deployment potential.

## 7. Conclusions and Limitations

### 7.1. Conclusions and Implications for Risk-Management Practice

This study develops EntropyFusion-VaR for tail-risk forecasting in three U.S. energy-sector equities: EOG, MPC, and SLB. The model combines entropy-state-driven allocation between temporal experts, a Risk-State Skip path, and structurally ordered quantile outputs. The manuscript’s benchmark summaries report lower average losses at the studied tail probabilities, while coverage is evaluated separately through exception frequencies and backtests. The contribution is a forecasting framework with component-level diagnostics for the selected stocks and test period.

The entropy inputs describe complementary aspects of ordinal complexity, overall distributional concentration, and directional predictive information. The displayed ablations show mean relative loss deterioration of about 8.3% without entropy features and 6.5% with fixed gating. Rényi entropy is useful here as a distributional-state descriptor, with its contribution evaluated empirically rather than assumed to be tail-specific. The output architecture has a displayed crossing rate of 0.00%. Across the nine stock–horizon combinations, EntropyFusion-VaR ranks first eight times and second once, with the lowest mean rank at each horizon. These results connect each main contribution to its corresponding loss, ordering, or ranking evidence.

For risk management in the studied equities, jointly ordered quantiles can support consistent risk-boundary monitoring. Dynamic gate weights provide an auxiliary view of changes in expert allocation and can be inspected alongside volatility, drawdown, and loss diagnostics. Their role is descriptive and predictive. Decisions about capital, hedging, or portfolio drawdown control require separate economic evaluation beyond the present forecasting comparisons.

### 7.2. Discussion of Limitations

Although EntropyFusion-VaR achieves strong extreme-tail forecasting performance within the current empirical framework, the study remains subject to limitations in data coverage, external validity, and the economic interpretability of deep models. These limitations identify important directions for future research.

The empirical scope is three selected U.S. energy-sector stocks, rather than the full set of energy financial markets. Their different value-chain positions provide within-sector variation, but they do not establish external validity for energy futures, carbon markets, broad indices, or other countries. At the 1% level, the limited number of expected exceptions and the descriptive nature of averaged statistics also constrain precision. Larger asset panels and dependence-aware uncertainty estimates from matched forecast records are natural extensions.

First, potential survivorship bias arises in the sample selection and construction of common risk features. When extracting common cross-sectional risk signals such as those for the S&P 500 market and the equal-weighted energy sector, this study relies primarily on representative constituents that remain in existence today and cannot fully incorporate data on tail firms that went bankrupt, were delisted, or underwent mergers and restructuring during extreme historical crises such as the initial outbreak of the pandemic in 2020. This survivorship bias may cause the training process to underestimate, to some extent, the absolute intensity of systematic market-wide tail risk and may create pressure on extreme-value calibration when the model confronts an unprecedented systemic collapse.

Second, the present study remains limited in the dimensionality of its data. Energy-asset prices are profoundly affected by geopolitical conflicts, OPEC+ production decisions, and macroeconomic monetary policy, whereas the current model relies primarily on price-volume time-series data and the associated physical entropy-complexity measures and does not directly incorporate unstructured alternative data. Future research could use large language models to extract news-sentiment factors and features from macroeconomic policy texts, or integrate satellite-based supply-chain data to construct a multimodal extreme-value forecasting network, thereby further improving the model’s sensitivity and forward-looking responsiveness to abrupt external shocks.

Finally, there remains scope to improve the theoretical interpretability of the deep-network architecture. The feature importance, gate-weight analysis, and component ablations provide partial transparency, while the nonlinear mappings learned inside the two experts retain black-box characteristics. Transfer entropy measures predictive information flow under the chosen conditioning scheme and does not, by itself, identify structural causality. Future research could evaluate the stability of feature attributions and expert allocations across stress regimes and incorporate economically justified structural restrictions. Such extensions would require separate validation; the present evidence supports predictive usefulness and limited component-level interpretation.

Further work could provide more detailed stock-specific coverage diagnostics across tail probabilities and market regimes, assess sensitivity to entropy-estimation and coverage-calibration settings, and compare overlapping and non-overlapping multi-day forecast designs using evaluation procedures that account for temporal dependence. These extensions would further characterize the stability and practical applicability of the framework across assets, parameter choices, and forecast horizons.

## Figures and Tables

**Table 1:** Dataset description and sample-period partitioning.

Dataset	Ticker	Representative Segment	Sample Split	Start Date	End Date	Total Trading Days
A	EOG	Upstream Exploration & Production	Train	4 January 2016	31 December 2020	1259
A	EOG	Upstream Exploration & Production	Validation	4 January 2021	31 December 2021	252
A	EOG	Upstream Exploration & Production	Test	3 January 2022	5 August 2026	1151
B	MPC	Downstream Refining & Marketing	Train	4 January 2016	31 December 2020	1259
B	MPC	Downstream Refining & Marketing	Validation	4 January 2021	31 December 2021	252
B	MPC	Downstream Refining & Marketing	Test	3 January 2022	5 August 2026	1151
C	SLB	Oilfield Services	Train	4 January 2016	31 December 2020	1259
C	SLB	Oilfield Services	Validation	4 January 2021	31 December 2021	252
C	SLB	Oilfield Services	Test	3 January 2022	5 August 2026	1151

**Table 2:** Summary of input variables and their economic meaning.

Feature Group	Variable Name (Variables)	Construction Method	Construction Window	Meaning/Role in the Model
Return	ret1	ln(Close_t_/Close_t−1_)	Daily	Core target-state signal
Absolute return	abs_ret	|ret1|	Daily	Shock magnitude
Squared return	ret2	ret1^2^	Daily	Nonlinear volatility proxy
High–Low range	hl_range	ln(High/Low)	Daily	Intraday risk
Overnight gap	overnight_gap	ln(Open_t_/Close_t−1_)	Daily	Overnight information shock
RV5	rv_5	5-day rolling SD × √252	5	Short-run volatility
RV20	rv_20	20-day rolling SD × √252	20	Monthly volatility state
RV60	rv_60	60-day rolling SD × √252	60	Persistent volatility state
Downside semivariance	downside_semivar_20	Mean[min(ret,0)^2^]	20	Downside risk intensity
Drawdown	drawdown_60	Close/max_60_ −1	60	Loss-state persistence
Market return	Market_ret1	Equal-weight S&P500 panel return	Daily	Systematic market context
Energy-sector return	EnergySector_ret1	Equal-weight energy-sector return	Daily	Sector-wide shock
Stock WPE	Stock_WPE_20/60/120	Weighted permutation entropy, m = 3	20/60/120	Firm information complexity
Market WPE	Market_WPE_20/60/120	Weighted permutation entropy, m = 3	20/60/120	Market complexity
Energy WPE	EnergySector_WPE_20/60/120	Weighted permutation entropy, m = 3	20/60/120	Sector complexity
Rényi-2 entropy	Renyi2_60/120	Rényi entropy, q = 2	60/120	Distributional concentration
Transfer entropy	TE_Market/Energy_to_Stock_126	First-order discretized TE	126	Directed information flow
Target return	target	Forward cumulative log return	h = 1/5/10	Quantile/VaR target

**Table 3:** Descriptive statistics for returns and the main multi-scale entropy features (based on the full sample).

Dataset	Stock	Variable	N	Mean	SD	Skew	Kurt	AC(1)	Min	Max
A	EOG	*Return*	2662	0.0004	0.0250	−1.32	27.94	−0.023	−0.3858	0.1533
A	EOG	*WPE20*	2662	0.8938	0.0727	−1.03	4.31	0.871	0.4133	0.9988
A	EOG	*WPE60*	2662	0.9636	0.0367	−3.46	19.11	0.966	0.6217	0.9990
A	EOG	*WPE120*	2662	0.9760	0.0352	−3.65	16.93	0.991	0.7566	0.9995
A	EOG	*TE Energy→Stock*	2662	0.1809	0.0163	1.09	4.21	0.997	0.1538	0.2320
A	EOG	*TE Market→Stock*	2662	0.1451	0.0125	1.04	4.05	0.997	0.1238	0.1838
B	MPC	*Return*	2662	0.0008	0.0258	−0.89	18.57	−0.037	−0.3148	0.1875
B	MPC	*WPE20*	2662	0.8887	0.0732	−0.86	3.46	0.882	0.5893	0.9988
B	MPC	*WPE60*	2662	0.9615	0.0314	−1.72	7.08	0.962	0.8046	0.9996
B	MPC	*WPE120*	2662	0.9798	0.0160	−1.73	7.29	0.972	0.8679	0.9997
B	MPC	*TE Energy→Stock*	2662	0.1598	0.0249	0.27	3.16	0.998	0.0841	0.2240
B	MPC	*TE Market→Stock*	2662	0.1279	0.0196	0.20	3.05	0.998	0.0672	0.1769
C	SLB	*Return*	2662	−0.0000	0.0254	−0.74	17.60	−0.010	−0.3205	0.1816
C	SLB	*WPE20*	2662	0.8889	0.0772	−1.16	4.88	0.888	0.4094	0.9987
C	SLB	*WPE60*	2662	0.9634	0.0326	−2.05	9.75	0.960	0.7135	0.9994
C	SLB	*WPE120*	2662	0.9789	0.0229	−2.62	13.09	0.986	0.8103	0.9999
C	SLB	*TE Energy→Stock*	2662	0.1729	0.0228	0.69	4.22	0.999	0.1115	0.2414
C	SLB	*TE Market→Stock*	2662	0.1386	0.0178	0.60	4.16	0.999	0.0886	0.1913

Note: N is sample size; SD is standard deviation; Skew and Kurt denote sample skewness and kurtosis; AC(1) is first-order autocorrelation. Min and Max are sample minima and maxima.

**Table 4:** Three-stock average forecasting and backtesting performance for 1% VaR across benchmark models.

Model	1%Pinball	Number of Exceptions	VR/CE (%)	K/CC/DQ/*p*-Value	Rank
EntropyFusion-VaR	0.716	11.6	1.01/0.01	0.828/0.669/0.429	1
TCN+Patch-Fixed	0.754	11.3	0.98/0.02	0.243/0.074 */0.211	2
Patch Transformer-QR	0.761	11.9	1.03/0.03	0.254/0.148/0.197	3
TCN-QR	0.775	12.1	1.05/0.05	0.107/0.087 */0.144	4
Q-LightGBM	0.787	12.5	1.09/0.09	0.252/0.202/0.331	5
LSTM-QR	0.793	12.9	1.12/0.12	0.304/0.215/0.206	6
GJR-GARCH-t	0.822	14.3	1.24/0.24	0.165/0.191/0.183	7
QRF	0.829	10.7	0.93/0.07	0.398/0.225/0.111	8
EWMA-Normal	0.851	10.4	0.90/0.10	0.175/0.225/0.358	9
CAViaR-AS	0.872	11.0	0.96/0.04	0.185/0.081 */0.119	10
HS-500	0.903	9.0	0.78/0.22	0.172/0.088 */0.216	11

Note: K/CC/DQ denote the Kupiec POF, Christoffersen conditional coverage, and dynamic-quantile tests, respectively. * *p* < 0.10. In VaR backtesting, stars indicate rejection of the corresponding null hypothesis of correct specification; therefore, more stars do not imply better model performance. Cross-stock average *p*-values are provided only as descriptive summaries. Pinball losses are reported in units of ×10^−3^.

**Table 5:** Three-stock average forecasting and backtesting performance for 2.5% and 5% VaR across benchmark models.

Model	Q.025 Loss	Q.025 VR/CE (%)	Q.025 Average K/CC *p*-Value	Q.05 Loss	Q.05 VR/CE (%)	Q.05 Average K/CC *p*-Value	Average Crossing Rate (%)	Rank 2.5%/5%
EntropyFusion-VaR	1.113	2.54/0.10	0.600/0.609	1.546	4.96/0.12	0.531/0.513	0.00	1/1
TCN+Patch-Fixed	1.195	2.47/0.13	0.403/0.094 *	1.668	4.91/0.15	0.340/0.363	0.33	2/3
TCN-QR	1.222	2.49/0.11	0.452/0.223	1.718	4.86/0.20	0.376/0.217	0.49	3/5
Patch Transformer-QR	1.231	2.43/0.12	0.256/0.390	1.652	4.88/0.16	0.534/0.240	0.39	4/2
LSTM-QR	1.252	2.51/0.14	0.531/0.145	1.704	4.97/0.18	0.382/0.160	0.56	5/4
Q-LightGBM	1.281	2.58/0.18	0.192/0.201	1.756	5.18/0.25	0.080 */0.102	0.70	6/6
GJR-GARCH-t	1.337	2.55/0.15	0.362/0.217	1.824	5.02/0.17	0.312/0.263	0.00	7/7
QRF	1.349	2.48/0.13	0.215/0.287	1.835	5.09/0.19	0.325/0.308	0.00	8/8
EWMA-Normal	1.385	2.61/0.19	0.278/0.261	1.891	4.94/0.20	0.514/0.119	—	9/9
CAViaR-AS	1.421	2.46/0.17	0.253/0.258	1.944	5.14/0.26	0.287/0.125	0.16	10/10
HS-500	1.466	2.67/0.23	0.161/0.195	2.010	5.22/0.29	0.156/0.204	0.00	11/11

Note: K/CC denote the Kupiec POF and Christoffersen conditional coverage tests, respectively. * *p* < 0.10. In VaR backtesting, significance stars indicate rejection of the corresponding null hypothesis of correct specification. Pinball losses are reported in units of ×10^−3^. Crossing rates reproduce Figure 6D as one joint-quantile summary. “—“ means EWMA-Normal is not reported in that panel.

**Table 6:** Correlation analysis between the TCN local-expert gating weight and market conditions.

Dataset	Ticker	External Market State/Signal	Spearman *ρ* (TCN/Local)	*p*-Value
A	EOG	Stock_WPE_120	0.220 ***	<0.001
A	EOG	EnergySector_WPE_60	0.110 ***	0.006
A	EOG	TE_Energy_to_Stock	−0.030	0.314
A	EOG	RV20	0.101 ***	<0.001
A	EOG	Drawdown60	−0.067 **	0.024
B	MPC	Stock_WPE_120	0.302 ***	<0.001
B	MPC	EnergySector_WPE_60	0.185 ***	<0.001
B	MPC	TE_Energy_to_Stock	−0.160 ***	<0.001
B	MPC	RV20	0.081 ***	0.006
B	MPC	Drawdown60	−0.094 ***	0.002
C	SLB	Stock_WPE_120	0.058 **	0.048
C	SLB	EnergySector_WPE_60	0.125 ***	<0.001
C	SLB	TE_Energy_to_Stock	−0.095 ***	0.001
C	SLB	RV20	0.194 ***	<0.001
C	SLB	Drawdown60	−0.052 *	0.079

Note: Significance markers following the Spearman correlation coefficients are * *p* < 0.10, ** *p* < 0.05, and *** *p* < 0.01. Stars indicate the statistical significance of the correlation.

**Table 7:** Stock-specific relative loss deterioration in the ablation experiments.

Ablation Variant	EOG (%)	MPC (%)	SLB (%)	Mean Displayed Deterioration (%)
Full	0.0	0.0	0.0	0.0
No-Calibration	2.1	1.1	1.9	1.7
No-Renyi	2.7	0.9	2.4	2.0
No-EntropicRobustness	4.0	1.3	3.5	2.9
No-RiskSkip	4.2	3.8	3.6	3.9
No-Patch Transformer	5.4	4.1	4.7	4.7
No-WPE	6.3	4.1	5.5	5.3
No-Transfer Entropy	7.1	4.1	6.2	5.8
No-TCN	6.6	6.0	5.8	6.1
Fixed-Gate	7.3	5.8	6.3	6.5
No-Entropy	9.6	7.1	8.3	8.3

Note: Stock-specific values reproduce Figure A2. The final column is their arithmetic mean, rounded to one decimal place. Positive values indicate higher loss relative to the full model.

**Table 8:** Relative loss comparison of the entropic and calibration components.

Loss Specification	Ablation Variant	EOG (%)	MPC (%)	SLB (%)	Mean (%)
Mean pinball plus calibration	No-EntropicRobustness	4.0	1.3	3.5	2.9
Entropic pinball without calibration	No-Calibration	2.1	1.1	1.9	1.7
Entropic pinball plus calibration	Full	0.0	0.0	0.0	0.0

Note: Percentages reproduce the corresponding variants in Figure A2 and Table 7. Means use the displayed values. The mean-loss variant removes the entropic component while retaining calibration.

**Table 9:** Cross-stock summary of model rankings across forecast horizons.

Model	Mean Rank h = 1	Mean Rank h = 5	Mean Rank h = 10	First-Place Finishes out of 9
EntropyFusion-VaR	1.00	1.00	1.33	8
TCN+Patch-Fixed	2.33	2.33	2.67	0
Patch Transformer-QR	3.67	3.00	2.00	1
TCN-QR	3.00	5.00	5.67	0
LSTM-QR	5.00	4.00	4.00	0
Q-LightGBM	6.00	5.67	5.33	0
HS-500	7.00	7.00	7.00	0

Note: Mean ranks average the three stock-specific ranks displayed in Figure A1. Lower ranks are better. First-place finishes are counted over the nine stock–horizon combinations. Only models displayed in Figure A1 are included.

**Table 10:** Comparison of model parameter counts and computational efficiency.

Model (Architecture)	Number of Trainable Parameters	Fitting Time per Dataset	Single-Step Inference Latency	Complexity/Efficiency Description
GJR-GARCH-t	18	0.42 s	0.008 ms	Very light parametric
CAViaR-AS	12	0.31 s	0.006 ms	Very light quantile regression
QRF	—	1.85 s	0.220 ms	150-tree forest
Q-LightGBM	—	0.94 s	0.080 ms	300 boosting rounds
LSTM-QR	64,611	3.25 s	0.055 ms	Single recurrent encoder
TCN-QR	52,227	2.75 s	0.040 ms	Dilated convolutions
Patch Transformer-QR	73,107	4.20 s	0.068 ms	2-layer patch attention
TCN+Patch-Fixed	81,000	5.10 s	0.092 ms	Fixed dual-expert fusion
EntropyFusion-VaR	82,709	5.65 s	0.105 ms	Dynamic entropy gate; favorable accuracy/size trade-off

Note: All computation times in the table are reported for a CPU environment; for the deep-learning models, batch size = 64 and the sequence input window is *L* = 120.

## Data Availability

The supporting dataset and code for this study can be downloaded at: https://zenodo.org/records/21871945 (accessed on August 9 2026). The original raw data were collected and integrated from publicly available databases, specifically the Kaggle database “S&P 500 Stocks (daily updated)”.

## Abbreviations

Abbreviation	Full Form
CC	Conditional Coverage
DQ	Dynamic Quantile
DRO	Distributionally Robust Optimization
EOG	EOG Resources
ES	Expected Shortfall
HS	Historical Simulation
IND	Christoffersen independence test
LSTM	Long Short-Term Memory
MPC	Marathon Petroleum
OOS	Out-of-Sample
POF	Kupiec unconditional coverage test
RE	Rényi Entropy
SD	Standard Deviation
SLB	Schlumberger
TCN	Temporal Convolutional Network
TE	Transfer Entropy
VaR	Value at Risk
WPE	Weighted Permutation Entropy

## Appendix A

**Table A1:** Hyperparameter and estimation settings for benchmark models and EntropyFusion-VaR.

Model	Category	Final Hyperparameters/Configuration	Training/Estimation	Validation/Test Rule
HS-500	Historical Simulation	Rolling window = 500; α = 1%, 2.5%, 5%	Empirical quantile of the previous 500 daily returns	No validation tuning; rolling window updated only in test
EWMA-Normal	Parametric volatility	λ = 0.94; Gaussian innovation	Recursive EWMA conditional variance mapped to VaR	Fixed specification; no test-period retuning
GJR-GARCH-t	Econometric	GJR-GARCH(1,1); Student-t innovation; constant mean	Maximum-likelihood estimation on training sample; recursive OOS forecast	Order/distribution fixed before test
CAViaR-AS	Dynamic quantile	Asymmetric Slope; VaR lag = 1; separate positive/negative return shocks	Quantile-loss minimization separately for each α	Validation used for convergence check only
QRF	Machine learning	n_estimators = 150; max_depth = 8; min_samples_leaf = 10; max_features = sqrt	Bootstrap forest; conditional leaf distribution used for quantiles	Validation specification fixed before test
Q-LightGBM	Machine learning	n_estimators = 300; learning_rate = 0.03; num_leaves = 31; min_child_samples = 20; feature_fraction = 0.8	Quantile objective; early stopping = 30 rounds	Validation selects effective boosting rounds
LSTM-QR	Deep learning	L = 120; hidden = 112; layers = 1; dropout = 0.10; batch = 64	Adam; lr = 1 × 10^−3^; max epochs = 200; patience = 15	Best validation epoch retained
TCN-QR	Deep learning	L = 120; channels = 32/32/64/64; kernel = 3; dilations = 1/2/4/8; dropout = 0.10	Causal dilated residual blocks; Adam; lr = 1 × 10^−3^; batch = 64	Validation early stopping; test used only for evaluation
Patch Transformer-QR	Deep learning	L = 120; patch = 10; d_model = 64; heads = 4; layers = 2; FFN = 128; dropout = 0.10	Adam; lr = 1 × 10^−3^; batch = 64; patience = 15	Best validation epoch retained
TCN+Patch-Fixed	Structural baseline	Same TCN and Patch settings; fixed expert weights = 0.5/0.5	Parallel experts with static equal-weight fusion	Controls for the value of dynamic Entropy Gate
EntropyFusion-VaR	Proposed model	L = 120; TCN and Patch settings as above; entropy encoder hidden = 32; gate hidden = 16; τ = 0.02; batch = 64	Adam; lr = 1 × 10^−3^; non-crossing joint-quantile head; entropic robust objective + calibration	Validation selects epoch and τ; test untouched

Note: Deep-learning models use the same 120-day input window and the validation set for early stopping/model selection; the test set is reserved for final out-of-sample evaluation. The entropy inputs use WPE with m = 3 over 20-, 60-, and 120-day windows, Rényi entropy with q = 2 over 60- and 120-day windows, and first-order discretized TE from the market and energy sector to each stock over 126 days (Table 2). The risk-sensitivity coefficient is γ = 0.02, denoted by τ in this table and Figure 9; it is distinct from the WPE embedding delay. In Equation (17), λ is the calibration weight and δ is the sigmoid smoothing bandwidth. The smooth proxy is used for training, while hard exception indicators are used for out-of-sample coverage evaluation.
